# PseudoGA: cell pseudotime reconstruction based on genetic algorithm

**DOI:** 10.1093/nar/gkab457

**Published:** 2021-07-09

**Authors:** Pronoy Kanti Mondal, Udit Surya Saha, Indranil Mukhopadhyay

**Affiliations:** Human Genetics Unit, Indian Statistical Institute, 203 B. T. Road, Kolkata 700108, West Bengal, India; Human Genetics Unit, Indian Statistical Institute, 203 B. T. Road, Kolkata 700108, West Bengal, India; Human Genetics Unit, Indian Statistical Institute, 203 B. T. Road, Kolkata 700108, West Bengal, India

## Abstract

Dynamic regulation of gene expression is often governed by progression through transient cell states. Bulk RNA-seq analysis can only detect average change in expression levels and is unable to identify this dynamics. Single cell RNA-seq presents an unprecedented opportunity that helps in placing the cells on a hypothetical time trajectory that reflects gradual transition of their transcriptomes. This continuum trajectory or ‘pseudotime’, may reveal the developmental pathway and provide us with information on dynamic transcriptomic changes and other biological processes. Existing approaches to build pseudotime heavily depend on reducing huge dimension to extremely low dimensional subspaces and may lead to loss of information. We propose PseudoGA, a genetic algorithm based approach to order cells assuming that gene expressions vary according to a smooth curve along the pseudotime trajectory. We observe superior accuracy of our method in simulated as well as benchmarking real datasets. Generality of the assumption behind PseudoGA and no dependence on dimensionality reduction technique make it a robust choice for pseudotime estimation from single cell transcriptome data. PseudoGA is also time efficient when applied to a large single cell RNA-seq data and adaptable to parallel computing. R code for PseudoGA is freely available at https://github.com/indranillab/pseudoga.

## INTRODUCTION

Cellular level gene expression profile can reveal the heterogeneity within a tissue and provides valuable information about ongoing biological processes inside a cell (1–4). In bulk RNA-seq data, averaging over large number of cells may hide the true biological signal coming from a heterogeneous mixture of cells. This phenomenon, commonly known as Simpson’s paradox, may give misleading conclusions. Biological processes like tissue development, cellular differentiation, tumor development, cell cycle etc. go through transcriptomic stages in cell specific manner. To understand the mechanism of the ongoing process it is essential to study the transcriptomic signature that triggers and controls these programmed changes (5). There is an underlying order (6–8) behind these transcriptomic stages that remains unexplored mainly due to the collection of cells at a single time point and inability to track the function over time. Clearly, not all cells are at the same stage during a biological process leading to cell to cell variability in gene expression profile. So capturing cells at a particular time would display different stages of cells that should be ordered according to a time scale, known as ‘pseudotime’.

Genes responsible for circadian rhythm, metabolism, cell death process etc. are regulated in a synchronized manner in different cells. Function of a cell may be affected by stages in development process, cell state transition, spatial effect, interaction with environment, cell–cell interaction and other internal ongoing processes. Effects of these simultaneous processes add to the heterogeneity in expression levels of thousands of genes at cellular level (9,10). Thus, arranging cells according to a pseudotime trajectory with respect to its transcriptional stages may provide more insight into the mechanism of how transcriptomic changes govern biological procedures at molecular level (3,11,12). This information might have important applications in therapeutics and system biology (6,13,14). The pseudotime need not be the physical time in a biological process; it could be a hypothetical time scale or pseudotime, depending on the developmental stage, position in cellular hierarchy, cell cycle stage and other biological processes.

Available methods in the literature mainly focus on dimensionality reduction followed by mapping of cells to a trajectory. The dimensionality reduction is performed by principal component analysis (PCA) (15), independent component analysis (ICA) (16), t-stochastic neighbor embedding (t-SNE) (17), diffusion map (DM) (18) or DDRTree (19). Pseudotime inference is based on reduced dimensional data instead of full data. After dimensionality reduction, few methods build minimal spanning tree (20,21), principal curve (22) or reverse graph embedding (19) to learn a principal tree from the data and creates a pseudotime path. Instead of following tree construction approach, diffusion pseudotime (23) ranks cells based on eigenvectors of the matrix whose elements follow Gaussian distributions with respect to euclidean distance between two cells and kNN graph is created using the diffusion map. scVelo (24) follows a different approach by inferring pseudotime based on the amount of pre-mRNAs and mature mRNAs present in a cell.

**Figure 1.**
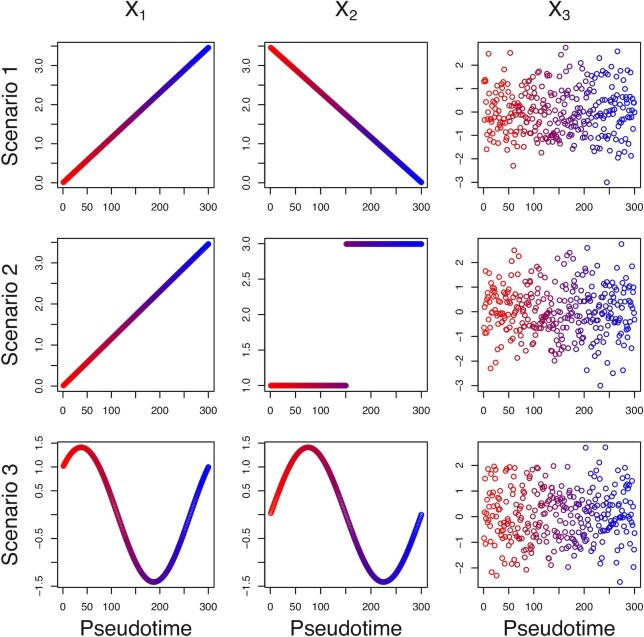
Three simulated scenarios each containing three variables (*X*_1_, *X*_2_, *X*_3_) having same variance with different types of trends. Scenario 1: *X*_1_ increasing, *X*_2_ decreasing and *X*_3_ random noise; Scenario 2: *X*_1_ increasing, *X*_2_ piecewise constant and *X*_3_ random noise; Scenario 3: *X*_1_ and *X*_2_ sinusoidal with phase difference, *X*_3_ random noise.

Existing pseudotime construction algorithms are mainly based on construction of minimal spanning tree, kNN graph or principal curve fitted on first two reduced dimensions. The accuracy of a method depends on the dimensionality reduction method being used in the first step and the amount of information that is lost during converting original data to lower dimensions. To check whether different types of dimensionality reduction algorithm can indeed construct the true pseudotime properly and retain most of the information that is in the original data, we simulate three dimensional data, under three scenarios (Figure 1). In each case, first two components are time dependent variables and all variables are scaled by standard deviation.

**Figure 2.**
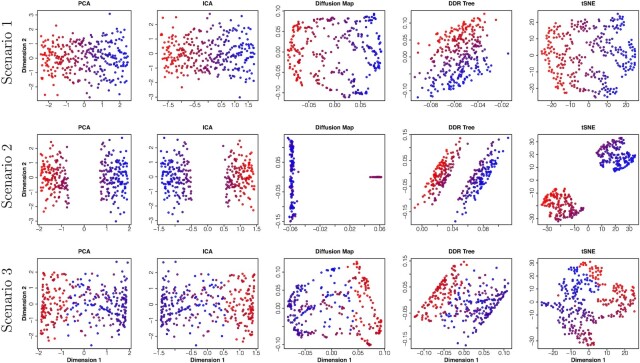
First two dimensions of outputs produced by PCA, ICA, diffusion map , DDRTree and t-SNE when applied to three scenarios as in Figure 1. Trajectory building algorithms based on minimal spanning tree, kNN graph or principal curve based on reduced dimensional data may not always retrieve the accurate behavior of actual pseudotime as the geometric patterns of the low dimensional space often do not truly reflect change in pseudotime.

We apply different algorithms for each scenario (Figure 2). In scenario 1, the first two variables are perfectly linear with pseudotime and the third variable is noise. First PCA and ICA components show linear trend with pseudotime. However, high variance for the second component adds more noise in its estimation while other dimensionality reduction methods do not show a clear picture of the pseudotime variable. In scenario 2, when there is a cascade like change in expression level of one variable, the pseudotime structure gets disrupted, though all methods show good characteristic of clustering. In scenario 3, both the variables are sinusoidal with phase difference. All dimensionality reduction methods fail to provide a clear picture of the temporal structure of the data. In all these three simulations, scatter plots of first two dimensions after applying dimensionality reduction techniques do not necessarily show visibly clear pattern of change in cell states along pseudotime. Certain trajectory reconstruction methods may fail to estimate approximate pseudotime values from some of these low-dimensional representations.

Our simulation shows that dimensionality reduction techniques may not always capture the full information about the pseudotime trajectory especially when few genes behave typically, like piece-wise linear etc. This simulation makes it clear that any method that is directly based on the actual gene expression values would have a higher chance to use more information and might provide more efficient and robust pseudotime ordering. We propose a novel method for pseudotime ordering of cells that is directly based on actual gene expression levels. Our method ‘PseudoGA’ uses genetic algorithm to come up with a best possible trajectory of cells that explains expression patterns for individual genes. Another advantage of this method is that it can identify any lineage structure or branching while constructing pseudotime trajectory.

## MATERIALS AND METHODS

For pseudotime estimation we apply genetic algorithm, which is appropriate for the current problem, to develop ordering of cells in the entire cell population. If the lineage structure or the branching between cell populations is of interest in addition to pseudotemporal ordering, cells are clustered into homogeneous subpopulations before applying the algorithm. The subpopulation structure can also be provided as input. Next, we apply the same algorithm to construct ordering of cells within same cell types. Finally, another subroutine concatenates the ordered paths from different clusters to form a tree like structure. Another highlight of our method is that our pipeline produces an undirected tree, connecting the paths from each cluster when no information on root cell is available. However, if the root cell or the cluster is identified or specified, our algorithm would provide an ordered tree. No transformation or dimensionality reduction is used in the pseudotime estimation step. We utilize full information from gene expression values within cells. However, if the lineage or branching structure is not of interest, the entire cell population is considered as one single subpopulation.

### Pseudotime ordering of cells

**Figure 3.**
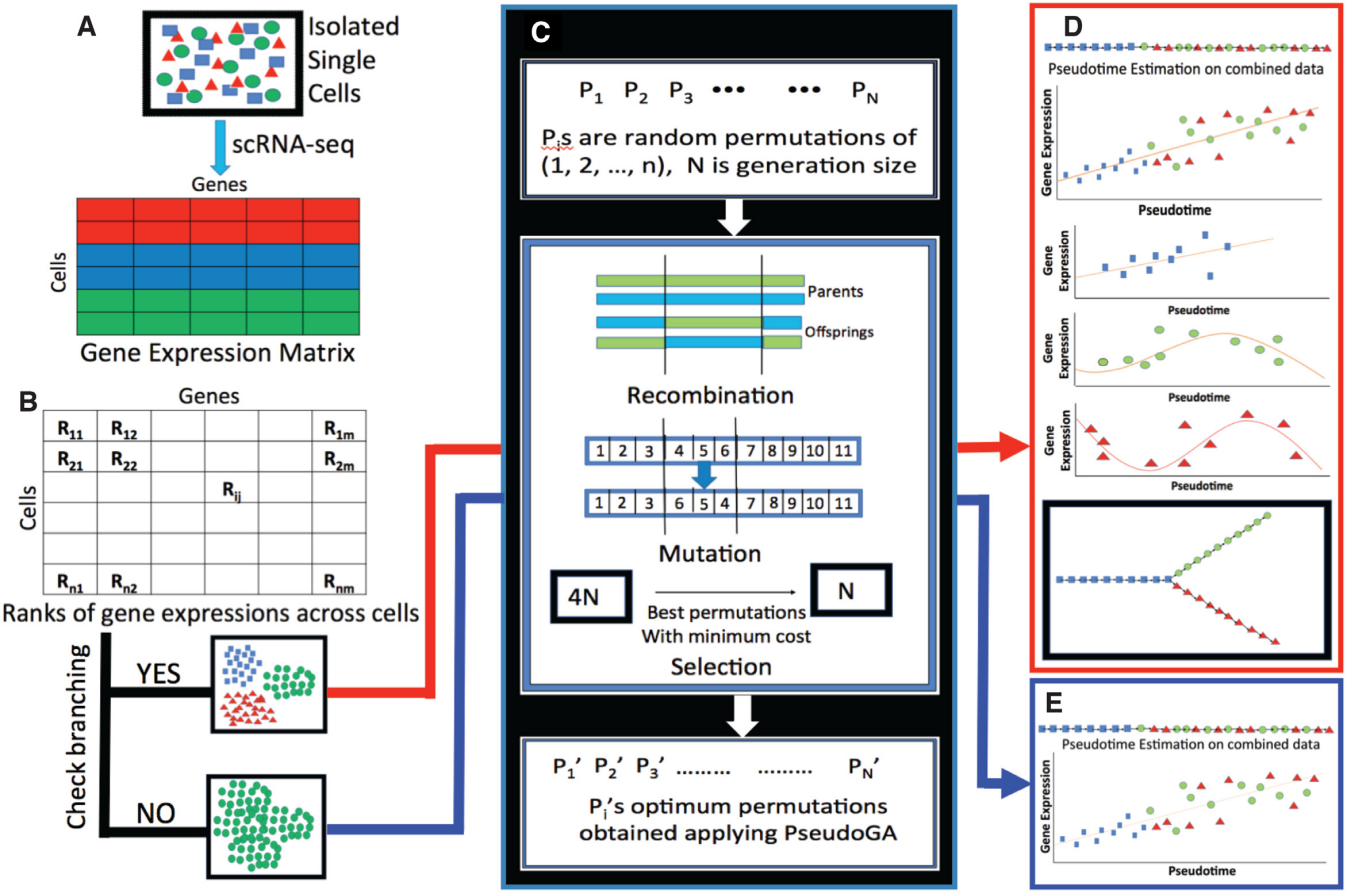
Outline of PseudoGA algorithm. (**A**) Single cell transcription profiles are used as input data. (**B**) Expression matrix is transformed into ranks of individual genes across cells. To check branching, cells are clustered based on expression profiles into homogeneous groups of cells; otherwise keep the entire dataset. (**C**) PseudoGA algorithm is applied to each cluster or full dataset. The solution space is the set of all possible ordering of cells. A group of candidate solutions is considered as a population. Starting with an initial population, the population is made to go through recombination, mutation and selection to arrive at improved solutions. (**D**) Based on pseudotime of individual clusters, behaviors of gene expression profiles are examined. Paths from different clusters are combined to make joint inference for the entire data. (**E**) Creates one pseudotime trajectory based on full data.

Data generated from a single cell whole transcriptome sequencing can be represented in a matrix *S* = ((*s*_*ij*_)) where *s*_*ij*_ is the gene expression corresponding to *i*th cell and *j*th gene (Figure 3A). Since expressions of all genes do not depend on pseudotime, a preliminary gene filtering is recommended to improve the accuracy of estimation. Cells are clustered optimally in at least two clusters. For pseudotime estimation, we select the top genes, that are differentially expressed between clusters. We can perform this step without clustering cells using variety of approaches like selection of highly variable genes (25,26), exploring relation between coefficient of variation and mean expression level (27,28), dropout-based feature selection (29) etc. However, application of our method to the entire dataset also produces similar results. Based on the expression levels of many genes together in a collection of cells, our objective is to place each cell on a certain time point to create a pesudotime trajectory. Most often, the use of trajectory inference in the analysis of single cell transcriptome data is reliant only on the ordering of cells and not on absolute values of the positions of cells on the trajectory. Quantitative positions on pseudotime trajectory may have no physical interpretation at all e.g. in cellular hierarchy data. Moreover, in reality, even if physical interpretation of values on pseudotime trajectory exists, a distance metric on cellular expression profile may not directly scale with the stretch between those two cells in the process under consideration. So, in this work, we consider discrete trajectories by finding the best permutation of cells such that the permutation explains gene expression level changes across transcriptome along the corresponding trajectory. The extent to which a pseudotime trajectory interprets specific changes in gene expression level can also be described in terms of a cost function. This cost or penalty is obtained by fitting a smooth curve with the expression values as a dependent variable and the pseudotime values as the explanatory variable. It may be noted that the problem of finding the best fitted pseudotime is similar to traveling salesman problem (TSP) (30). Here also given a complete undirected graph with certain edge weights, the problem is to find the Hamiltonian path with the shortest weight. The pseudotime problem we are dealing with is slightly different because the cost associated with a pseudotime path need not be the sum of costs between two consecutive cells. However, like TSP, the search space of our problem is the set of all permutations and we apply genetic algorithm to find a near optimal solution for any function defined on this space. Given the fact that the search space is discrete and grows exponentially with the number of cells, some heuristics is inevitable to find a near optimal solution. Genetic algorithm is known to perform reasonably well in a wide spectrum of problems (31,32) including the ones where the search space is the set of all permutations (33–39).

### Representation of ordering

Ordering of *n* objects can easily be represented by a permutation of natural numbers 1, 2, 3, …, *n*. Genetic algorithm (40) is a computational procedure that mimics biologically inspired operators such as mutation, crossover and selection to tackle the optimization problems (Figure 3C). It uses the idea of these biological phenomena in a computational or algorithmic paradigm and not in the actual biological sense. For example, a crossover in genetic algorithm generates a new list of permutations for evaluation in the next iteration, similar to a genomic crossover that generates a new set of markers on the chromosome. So, to apply genetic algorithm in an optimization problem, one first needs to find a suitable chromosomal representation (Supplementary Figure S1) of a candidate solution, using which genetic operators like mutation, recombination, and selection can run on the space of all possible solutions. In our work, we have used the permutation representation of ordering. We index the cells by 1, 2, …, *n* where 1st, 2nd,…, *n*th cells are chosen randomly. We represent a pseudotime ordering of cells indexed with *i*_1_, *i*_2_, …, *i*_*n*_ by the vector (*i*_1_, *i*_2_, …, *i*_*n*_) which is indeed a permutation of 1, 2, …, *n*. Since the chromosomal representation is only for computational purpose and has no biological significance, recombination, mutation and selection operators when applied on a permutation give birth to a new one that needs to be checked for a better solution.

### Cost function

Expression values of a gene over the pseudotime path may be a linear or nonlinear function of pseudotime. To make our proposed algorithm more general, we assume that the rank of the expression values over cells is a polynomial of pseudotime of degree at most 3 (Figure 3D, E). By using ranks instead of actual expression values (Figure 3B), we avoid the particular effect of any specific functional form of the gene expression, while retaining the general pattern. This non-parametric approach allows us to include a wide range of functional forms for gene regulation and also the outliers. There must be a tradeoff between number of parameters and degree of the polynomial that is used to fit the model. Since it is well established that some genes may behave in a cyclic manner with pseudotime, we allow the polynomial degree up to 3 for fitting the data. Our careful extensive inspection and other studies (20,21,41–45) observe that gene expression regulation along pseudotime usually reveals expression patterns mainly of three types: (i) expression that increases or decreases with time, (ii) expression that first increases and then decreases or vice versa and (iii) expression that first increases, then decreases, and then increases again with pseudotime, or vice versa. Our model can capture these three types of genes, assuming that ranks of gene expression values along pseudotime trajectory, can be either linear, quadratic or cubic function of the pseudotime. Mathematically, this model is:

$$\begin{equation*} R_{ij}=f_j(t_i)+\epsilon _{ij} \end{equation*}$$

where *R*_*ij*_ is the rank of cell *i* in the expression levels of gene *j*, *t*_*i*_ is the pseudotime for cell *i* and ε_*ij*_ is the random error term. *f*_*j*_ is an unknown function according to which gene expression changes over pseudotime. In our set up, *t*_*i*_ ∈ {1, 2, …, *n*}, for all *i* and {*t*_1_, *t*_2_, …, *t*_*n*_} is a permutation of {1, 2, …, *n*}. If we approximate *f*_*j*_ by a cubic polynomial, the regression equation becomes:

$$\begin{equation*} R_{ij}=\beta _{j0} + \beta _{j1}t_i + \beta _{j2}t_i^2+ \beta _{j3}t_i^3 + \epsilon _{ij} \end{equation*}$$

Let $\hat{\beta }_{jk}$ be the least square estimate of β_*jk*_, *k* = 0, 1, 2, 3, for a pseudotime ordering (*t*_1_, *t*_2_, *t*_3_, …, *t*_*n*_). Then, the cost associated with the ordering of *j*-th gene by cubic polynomial is given by Bayes Information Criterion: $\text{BIC}_{3j}=n\text{ln}(\sigma _{j\epsilon }^2)+3 \text{ln}(n)$ with

$$\begin{equation*} \sigma _{j\epsilon }^2=\frac{1}{n}\sum \limits _{i=1}^{n} (R_{ij}-\hat{\beta }_{j0}-\hat{\beta }_{j1}t_i-\hat{\beta }_{j2}t_i^2-\hat{\beta }_{j3}t_i^3)^2 \end{equation*}$$

Similarly, we define BIC_1*j*_ and BIC_2*j*_ as BIC values associated respectively with fitting linear and quadratic polynomial on the rank of expression values with pseudotime as explanatory variable. Now the cost associated with *j*-th gene for the given pseudotime is *C*_*j*_ = *min*(BIC_1*j*_,BIC_2*j*_,BIC_3*j*_). Overall cost associated with the pseudotime {*t*_1_, *t*_2_, …, *t*_*n*_} for the whole transcriptome expression profile is $C=\sum \limits _{j=1}^{n_G} C_j$ where *n*_*G*_ is the total number of genes. It is important to note that we treat the zero expressions in the data as numeric zeros and use them in ranking. If there are ties in expression values, we assign average rank to all observations with ties. The introduction of the cost function *f*_*j*_ adds more flexibility to our model. Any prior knowledge leading to more specific form of *f*_*j*_ can easily be incorporated in the model and the entire downstream protocol will follow accordingly. Naturally, this would result in more efficient estimation of pseudotime.

### Genetic algorithm for pseudotime construction

Let $\mathbb {X}$ be the space of all permutations of the set {1, 2, …, *n*}. The cost function *C* is a function $C:\mathbb {X}\rightarrow \mathbb {R}$, where $\mathbb {R}$ denotes the real line. *C* contains penalty incurred due to non-optimality of an ordering. Hence, the optimal pseudotime ordering is obtained by minimising *C*(*x*) with respect to *x* that moves in the space of all possible permutations *i.e*. $\mathbb {X}$. If *x*_*opt*_ is the optimal ordering, we have $x_{opt} = \arg \min \limits _{x\in \mathbb {X}} C(x)$. Since the solution space is discrete, standard useful analytical tools like continuity or differentiability cannot be applied to find an optimal solution. Hence, we apply genetic algorithm to find *x*_*opt*_. The algorithm uses the entire information from the dataset without any dimensionality reduction. Although it may not always find global optimality, it provides a reasonably good solution, at least, because no information is lost due to dimensionality reduction. Note that some other discrete optimisation algorithms may be used to address this problem. But we restrict to genetic algorithm and its modification tuned to this problem, mainly due to its wide applicability and better performance.

We consider three operators, mutation, recombination and selection in one single iteration. Mutation (Supplementary Figure S2) creates a new vector *y* from a given permutation *x* by randomly choosing two positions *i* and *j* with *i* ≤ *j* such that *y*_*k*_ = *x*_*k*_(1 − *I*_{*i* ≤ *k* ≤ *j*}_) + *x*_*i* + *j* − *k*_*I*_{*i* ≤ *k* ≤ *j*}_ where *I*_{.}_ denotes the indicator function. If *x*_*i*_ and *x*_*j*_ are the values of *x* at positions *i* and *j* respectively, after mutation the new values would be *y*_*i*_ = *x*_*j*_ and *y*_*j*_ = *x*_*i*_. This mutation operator is essentially an inversion (46) applied on a portion of the chromosome with two randomly chosen endpoints. Mutation adds *N* extra mutated individuals to an existing population of size *N*.

Several recombination or crossover operators on permutations have been suggested, e.g. partially mapped crossover (47), order crossover (48), cycle crossover (49) etc. We propose a recombination operator (Supplementary Figure S3) for this context that is similar to a partially mapped operator (47). First, the set of individuals is divided into two subpopulations of equal size. Crossing over occurs between pairs with one from each group. Instead of taking only two cut points as in partially mapped crossover, we consider the number of cut points to be a Poisson random variable. If the random number generated is *N*, (*N* + 1) fragments of equal length are formed from the parent string. Alternate fragments from one of the parents, say string I, are retained in one of the two newly formed offspring strings. To fill up the missing positions, the entries not retained in the newly formed string are recorded. A bipartite graph is constructed with the positional indices of those left behind entries of the two parent strings as vertices on two sides. All possible edges between vertices on the two sides are considered and the absolute differences between the ordinal position values are taken as edge weights. Based on the bipartite graph thus created, minimal bipartite matching is constructed. Entries in the positions considered in the other string, say string II, are put into the corresponding positions in string I based on the minimal bipartite matching graph. Using the same approach, another child is created by interchanging the role of string I and string II. Thus for an existing parent population of size *N*, same number of offsprings are added to it. It can be easily pointed out that the offsprings generated from this operation are not unique because the solution of minimal bipartite matching is not unique.

Mutation and recombination make a pool of 4*N* individuals from a pool of *N*. In the selection step (Supplementary Figure S4), only the top one quarter, i.e. top $\frac{N}{4}$ individuals with minimum cost, calculated on the basis of estimated cost function, are passed on to the next generation. This would keep the size of candidate solutions for *x* vector same in each iteration.

### Construction of branching and lineage by joining different clusters

Till now we consider applying our method to the entire dataset. However, if we want to see any existence of branching, first we have to cluster the data. PseudoGA will be applied on each cluster considering it as the full data. Once the pseudotime orderings within the clusters are formed, we can construct lineages assuming a continuum between clusters (Figure 3D). This is important in many applications like construction of developmental trajectory, detection of bifurcation, building cellular lineage etc. Note that, the ordering within each cluster has two termination points. The distances between termination points across clusters are computed using extreme cells at either side of the path. The distance between two paths $\vec{x}=(i_1,i_2,\ldots ,i_m)$ and $\vec{y}=(j_1,j_2,\ldots ,j_n)$ is defined as $d(\vec{x},\vec{y})=min(C(x_1),C(x_2),C(x_3),C(x_4))$ where

$$\begin{equation*} x_1=(j_{(\lfloor {\frac{n}{4}}\rfloor )},\ldots ,j_2,j_1,i_1,i_2,\ldots ,i_{(\lfloor {\frac{m}{4}}\rfloor )}), \end{equation*}$$

$$\begin{equation*} x_2=(j_{(\lfloor {\frac{3n}{4}}\rfloor +1)},\ldots ,j_{(n-1)},j_n,i_1,i_2,\ldots ,i_{(\lfloor {\frac{m}{4}}\rfloor )}), \end{equation*}$$

$$\begin{equation*} x_3=(i_{(\lfloor {\frac{m}{4}}\rfloor )},\ldots ,i_2,i_1,j_1,j_2,\ldots ,j_{(\lfloor {\frac{n}{4}}\rfloor )}), \mathrm{and} \end{equation*}$$

$$\begin{equation*} x_4=(i_{(\lfloor {\frac{3m}{4}}\rfloor +1)},\ldots ,i_{(m-1)},i_m,j_1,j_2,\ldots ,j_{(\lfloor {\frac{n}{4}}\rfloor )}), \end{equation*}$$

where *C*(*x*) is the cost function as defined before and the ‘floor’ function ⌊*x*⌋ denotes the greatest integer less than or equal to *x*.

A common approach to construct lineage from disjoint clusters of homogeneous populations of cells is by constructing minimal spanning tree (MST) on cluster centres (14,21,22). Here we adopt a similar approach. Following Kruskal’s algorithm for minimal spanning tree, the termination points with minimum distances are joined until a tree structure is constructed, taking into consideration that no cycle is formed. If multiple clusters join with a single termination point in a path, a new branching point is added near that termination point. In this way, we construct an undirected graph with tree like structure with branching points. If the purpose is to find a directed graph of clusters, the user would provide either a root cell or a root cluster. Now, a directed network is constructed using the root cluster and the undirected graph. In the HSMM dataset (19,50), three clusters have been observed while performing cluster-wise pseudotime estimation. The t-SNE plot applied on this data clearly shows a lineage structure (Supplementary Figure S5). The network between clusters by assuming cluster 2 as the root cluster is shown in Supplementary Figure S6. The network can be visualized with any low dimensional representation of the data. It is consistent with the overall structure of reduced dimensional representations produced by PCA, diffusion map and t-SNE (Supplementary Figure S7). In all these three embeddings, there is a transition from cluster 2 to a bifurcation into cluster 1 and cluster 3. Our pseudotime ordering agrees to the ordering with all three low dimensional embeddings. PseudoGA branching trajectory is very similar to the lineage produced by Monocle 2 (19) on the same dataset.


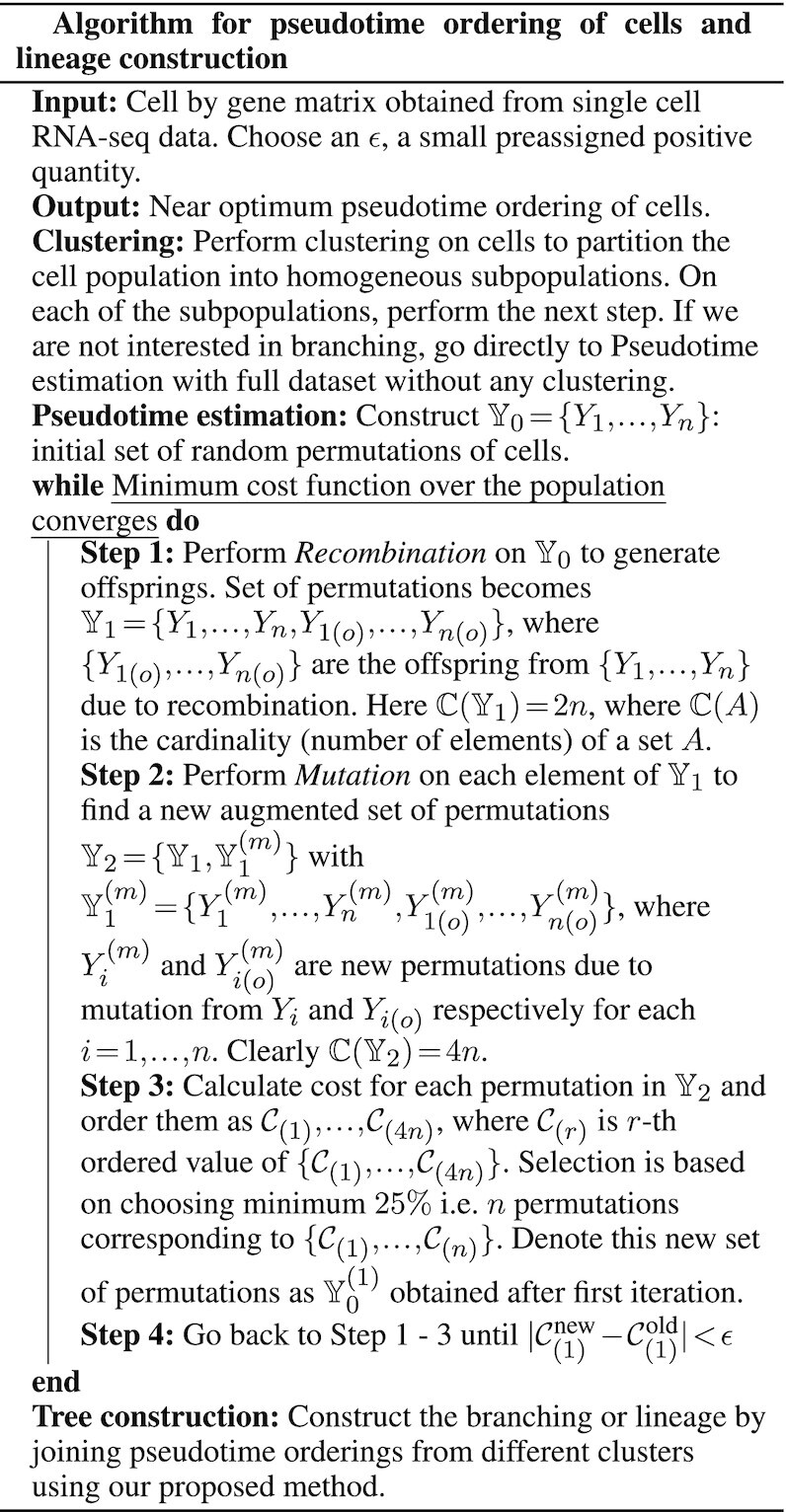


### Pseudotime estimation with large number of cells

Genetic Algorithm for finding optimal permutation scales poorly with number of cells. Some modification of our algorithm is required to construct pseudotime with large number of cells. First, we subsample a smaller number of cells and apply our proposed method.

Out of *N* total cells, pseudotime is estimated based on a subset of *n* (*n* < *N*) cells. Suppose (*t*_1_, *t*_2_, …, *t*_*n*_) is the vector of estimated pseudotime for *n* cells. We define a score for every cell *j*, $S_j=\frac{1}{r}\sum \limits _{k\in N_r(j)}t_k$ where *N*_*r*_(*j*) is the set of *r* nearest neighbors of cell *j*. The vector *S* = (*S*_1_, *S*_2_, …, *S*_*N*_) or the ordering of *S* can be considered as the pseudotime for the original set of *N* cells.

To increase the efficiency, instead of inferring trajectory based on one subset, one can consider pooled inference from multiple subsamples as well. Based on *B* (say 30) subsamples each of size *M* (say 100) from the same dataset, we construct pesudotime trajectories separately. We find score (*S*_*jb*_,  *b* = 1, …, *B*) of the *j*th cell corresponding to each *b*-th trajectory and construct a principal curve based on *B* dimensional scores of individual cells. The principal curve has been used for pseudotime reconstruction in different manners (22,51,52). In our algorithm, ordering of cells on the principal curve is the final pseudotime trajectory for the entire dataset. Naturally, larger the number of subsamples, more will be the accuracy. However, we observe that 30 subsamples would show a significant improvement in correlation (0.99) with actual pseudotime (Supplementary Figure S8). Inferring the final trajectory based on multiple estimates makes this approach robust to unwanted variation present in the data.

## RESULTS

We evaluate our method ‘PseudoGA’ and compare it to other methods using extensive simulations and five different real datasets including one that contains a large number of cells. In all datasets under consideration, we measure the accuracy using appropriate measures. Monocle (20), TSCAN (21), Slingshot (22), DPT (23), Waterfall (14) and scVelo (24) are used for comparison since they are all de novo pseudotime reconstruction techniques based on unique approaches and their open source codes are available. The benchmarking also indicates how different dimensionality reduction methods perform in constructing pseudotime trajectory. scVelo has been used for comparison on real data only because in synthetic gene expression datasets, expression values are directly simulated without mimicking exact RNA-seq experiment whereas scVelo requires raw reads for estimation.

### Pseudotime determination using real data

We consider five real datasets for benchmarking. We evaluate reference trajectories for all these datasets based on the given information like time of collection, stage etc. To compare precisions of different estimates, we use absolute Spearman’s rank correlation. Moreover, PseudoGA estimates can be visualized in low dimensional embeddings using any dimensionality reduction method including PCA. We also attempt to explore genes that are highly correlated with estimated pseudotime and whether they have any significance in the context of known actual pseudotime. We now briefly describe the real datasets and the interpretations of pseudotime for the concerned experiments.

**Figure 4.**
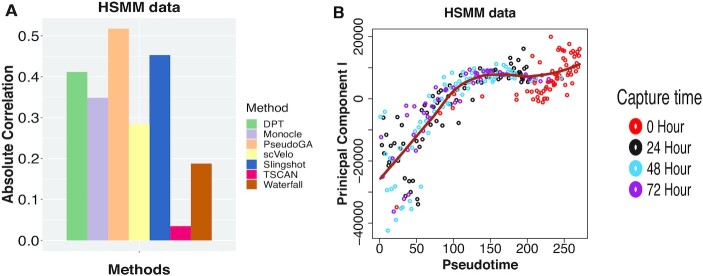
(**A**) Absolute Spearman’s rank correlation between HSMM data capture time and pseudotime produced by different methods for the entire dataset. PseudoGA shows the highest correlation among all methods. (**B**) Plot of PC I with pseudotime estimated by PseudoGA shows monotonically increasing pattern.

Skeletal myoblasts are set to undergo a well-characterized sequence of morphological and transcriptional changes during differentiation. Primary human skeletal muscle myoblasts (HSMM) were expanded under high mitogen conditions and then differentiated by switching to low mitogen media (GSE52529) (20,50). RNA-seq libraries were sequenced from each of several hundred cells taken over a time-course of serum-induced differentiation. Around 49 to 77 cells were captured at each of the four time points (0, 24, 48 and 72 h). The capture time here can be assumed to be the underlying pseudotime. First, we perform pseudotime analysis on the entire dataset. First principal component (PC) shows an increasing pattern with pseudotime estimated by PseudoGA (Figure 4B). Plot of PC II exhibits a parabolic pattern with respect to pseudotime (Supplementary Figure S9).

If we are interested to see any lineage or branch structure with respect to pseudotime, we have to cluster the original data and apply our algorithm on each cluster. Clustering with t-SNE creates three clusters with one cluster consisting of observations from 0 hours only (Cluster II), two other clusters (Cluster I and III) with mixture of observations from 24, 48 and 72 h (Supplementary Figure S11A). Cells from three different time points in cluster I are well separated whereas the cells from three populations in cluster III are mixed up. For visualisation, we plot PC I and PC II with respect to pseudotime as estimated by PseudoGA that show overall linear or quadratic trend in all these clusters ( Supplementary Figure S11B).

PseudoGA has the highest correlation among all methods under consideration when applied on the entire dataset (Figure 4A) although Slingshot performs slightly better when clusters are considered separately (Supplementary Figure S10). However, PseudoGA seems more robust because its performance is consistently good in all scenarios. We find top 6 genes having highest correlation with pseudotime for the whole HSMM dataset as well as for three clusters separately (Supplementary Tables S1 and S2) and observe the change of expression along pseudotemporal path (Supplementary Figures S12 and S13).

Our next dataset contains single cell RNA-seq data from 1861 mouse dendritic cells stimulated with three pathogenic components. This dataset is used to examine the variation between individual cells exposed to the same stimulus and study the complex dynamic responses to the stimulus exhibited by multicellular populations (GSE48968) (41). Cells were captured initially without any stimulus, and at one, two, four and six hours after applying the stimulus. Cell capture time in this case can be considered as the pseudotime. To compare accuracy of different methods, cells that were applied different types of stimuli, were sorted out. Three different types of stimuli, namely LPS, PAM and PIC were applied.

**Figure 5.**
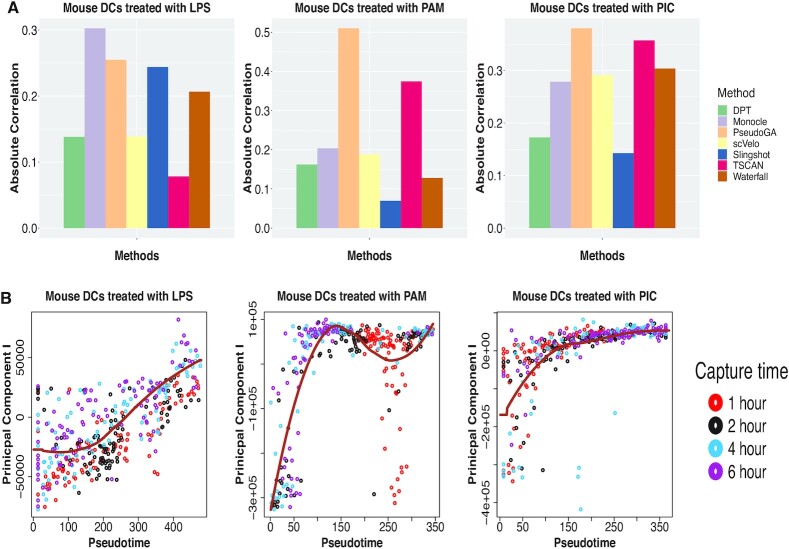
(**A**) Absolute Spearman’s rank correlation between capture time of dendritic cells and pseudotime produced by different methods for the entire dataset under stimulation by LPS, PAM and PIC. PseudoGA shows overall best performance. (**B**) Plot of PC I with pseudotime estimated by PseudoGA for three different types of simulation. In the stimulation by LPS, PC I shows linear pattern whereas in the other two datasets, PC I shows bursting type pattern with pseudotime.

We apply pseudotime estimation algorithms on all these three types of data with different stimuli. Application of PseudoGA on the entire data for each stimulus shows that estimated pseudotimes are in overall congruence with the actual pseudotime (Figure 5A). First two principal components show strong functional relationship with pseudotime estimated by PseudoGA (Figure 5B, Supplementary Figure S14). Under all these three types of stimuli, PseudoGA shows the best performance among these seven methods (Figure 5A). Only in the data for mice treated with LPS, Monocle performs better than PseudoGA although for other two datasets its performance is not really good. On the other hand, PseudoGA consistently shows high correlation and clear pattern with pseudotime for all stimuli. Top 6 genes having highest correlation with pseudotime in different clusters and their common functions are shown in Supplementary Tables S3–S5. The change of expressions with pseudotime along pseudotemporal path produced by PseudoGA in these three scenarios are shown in Supplementary Figures S15–S17.

**Figure 6.**
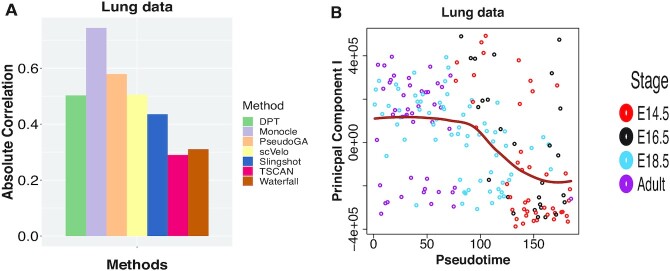
(**A**) Absolute Spearman’s rank correlation between developmental stage and pseudotime assigned by different methods on lung data. Monocle shows the highest correlation followed by PseudoGA. (**B**) Plot of PC I with pseudotime estimated by PseudoGA. PC I shows decreasing pattern with pseudotime though it can also be viewed as bursting.

The third dataset consists of microfluidic single cell RNA-seq on 185 individual mouse lung epithelial cells at four different stages (E14.5, E16.5, E18.5, adult) of development (GSE52583) (42). The transcriptome data present transcriptional states that define the developmental and cellular hierarchy of distal mouse lung epithelium. Cells were assigned to two groups, prenatal and postnatal cells. Here the developmental stage can be considered as the underlying pseudotime. The plot of PC I shows transcriptional bursting pattern whereas the plot of second principal component shows monotonic pattern (Figure 6B and Supplementary Figure S18).

Monocle shows the highest correlation with actual pseudotime whereas PseudoGA turns out to be second best (Figure 6A). Top 6 genes having highest correlation with pseudotime and their common functions are shown in Supplementary Table S6. The change of expressions with pseudotime along pseudotemporal path is shown in Supplementary Figure S19.

**Figure 7.**
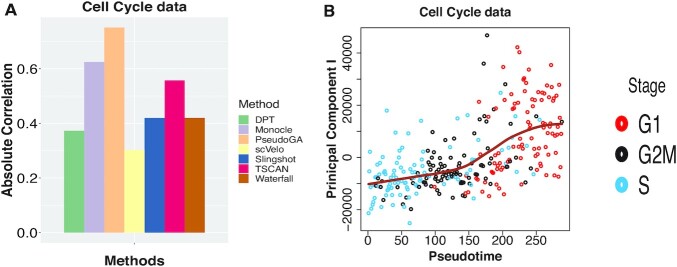
(**A**) Absolute Spearman’s rank correlation between developmental stage and pseudotime assigned by different methods on cell cycle data. Maximum correlation among all possible permutations of G1, S and G2M stages has been shown. PseudoGA again shows the highest correlation followed by Monocle. (**B**) Plot of PC I with pseudotime estimated by PseudoGA. The plot shows increasing pattern of PC I.

In the fourth dataset, we study the gene expression patterns at single cell level across three different cell cycle stages each containing 96 mouse embryonic stem cells (E-MTAB-2805) (26). Single cell RNA-seq was performed on mouse embryonic stem cells (mESC) that were stained with Hoechst 33342 Flow cytometry and sorted for G1, S and G2M stages of cell cycle. PseudoGA is able to separate cells with respect to G1, G2M and S stages from a mixture of cells. Thus it provides a potential ordering of cells across cell cycle. Only for visualization purpose, when we plot the first two PCs, it seems that PC I across pseudotime shows an increasing pattern whereas PC II indicates linear pattern (Figure 7B and Supplementary Figure S20).

Maximum correlation among all possible permutations of G1, S and G2M stages were considered in this case as the order of cell cycle can be rearranged. Here again, PseudoGA shows the highest correlation among all methods (Figure 7A). Top 6 genes having highest correlation with pseudotime and their common functions are shown in Supplementary Table S7. The change of expressions with pseudotime along pseudotemporal path in these three scenarios are shown in Supplementary Figure S21.

**Figure 8.**
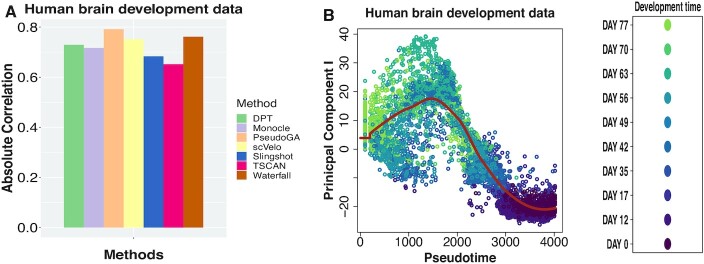
(**A**) Absolute Spearman’s rank correlation between developmental stage and pseudotime assigned by different methods on human brain development data. PseudoGA shows the highest correlation followed by Waterfall. (**B**) Plot of PC I with pseudotime estimated by PseudoGA. PC I changes as quadratic polynomial with respect to PseudoGA estimate.

We consider another dataset where gene expression profiles of human ventral midbrain cells were studied at different developmental stages after gestation ranging between 0 and 11 weeks (GSE76381) (53). This dataset is much larger than the four other datasets discussed earlier. Single cell RNA sequencing was performed on 4029 cells from different stages. So, the developmental stage of a cell can be considered as the inherent pseudotime in this case. We performed the pseudotime analysis on the entire dataset. PseudoGA estimate shows the highest correlation with the actual pseudotime (Figure 8A). The first two principal components show quadratic or cubic functional relationship with the pseudotime estimated by PseudoGA (Figure 8B, Supplementary Figure S22). Top 6 genes with highest correlation with the estimated pseudotime have either cubic or bursting type pattern (Supplementary Figure S23, Supplementary Table S8). This result establishes the consistency of performance as well as robustness of PseudoGA when applied to a large number of cells. However, it is to be noted that if we want to see any possible branching in pseudotime, we have to cluster the data and apply our algorithm on each cluster which afterwards would be merged to give a consolidated structure.

### Pseudotime using simulated data

We simulate datasets to evaluate different methods and compare their performance to PseudoGA. Simulations were performed with two different frameworks: simulation with our own simulation model and three other simulation schemes available in Bioconductor package ‘Splatter’ (57). Our simulation model can generate single-cell level expression profiles with known inherent pseudotime within a homogeneous population.

Note that all genes are not expressed in all cells. So, conditional on the fact that a gene is expressed in a cell, the read count corresponding to this gene is generated from a Poisson distribution whose mean follows a Gamma distribution. The shape parameter of the Gamma distribution for each expressed gene lies on a pseudotime curve. If a gene is not expressed in a cell, we consider its read count to be identically equal to zero. We consider different gene sets where a particular trend is being followed for a set. Thus expression values within a given set may follow increasing or decreasing linear trend, quadratic or sinusoidal trend. To add more generality, we also consider few genes whose expressions are independent of pseudotime.

We know that the abundance of technical zeros or dropouts (54,55) is a common feature in single cell RNA-seq data. So, we also add zero values for gene expressions that would naturally inflate the left tail of the Gamma-Poisson distribution. Regarding generation of dropouts, we consider three different scenarios. In the first case, we introduce lower rate of dropouts that are mainly due to smaller mean gene expression levels whereas in the second scenario, it is independent of mean expression values. In the third case, we assume relatively higher amount of dropouts that occur independently of pseudotime. Detailed algorithm for simulation is described in Supplementary Section 1.

Note that if a gene regulation has a prominent relation with pseudotime, it would show a trend, at least approximately. In order to capture this trend, it is expected that enough information should be available for that gene. Hence it is natural to believe that the amount of technical zeros, which is a common characteristic of single cell data, should be relatively small.

So the first two scenarios are more rational when the dataset is of good quality or genes with lower dropout rates are filtered successfully before pseudotime estimation. Scenario III is relevant when the data contain too many technical zeros and in addition to that, either gene filtering cannot separate out genes with lower dropout rates or no filtering is applied.

We apply PseudoGA and other commonly available methods to these simulated datasets. Entire study is based on 100 replicates under each simulation scheme. We assess the accuracy of each method using two criteria, (i) absolute rank correlation coefficient between estimated pseudotime with the actual one and (ii) number of genes that show functional relationship with the estimated pseudotime.

**Figure 9.**
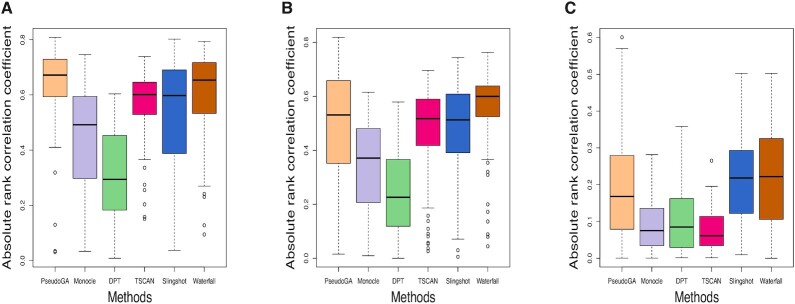
Absolute correlation coefficient with the actual pseudotime in (**A**) Scenario 1: lower dropout rate and dropout probability depends on mean expression level, (**B**) Scenario 2: lower dropout rate and dropout probability is independent of mean expression level, and (**C**) Scenario 3: higher dropout rate and dropout probability is independent of mean expression level. PseudoGA shows overall consistent performance across all three scenarios.

**Figure 10.**
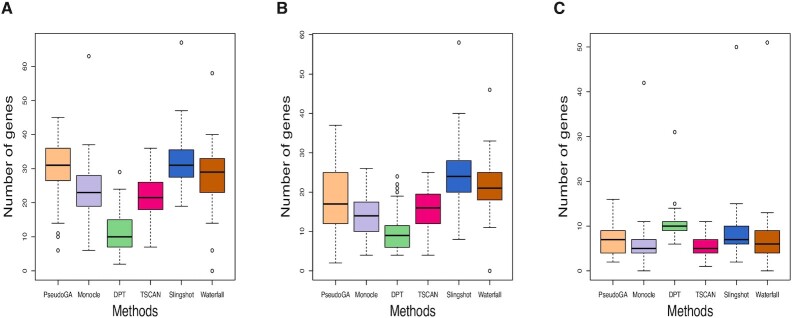
Number of genes that have functional relationship with the pseudotime estimated in (**A**) Scenario 1: lower dropout rate and dropout probability depends on mean expression level, (**B**) Scenario 2: lower dropout rate and dropout probability is independent of mean expression level and (**C**) Scenario 3: higher dropout rate and dropout probability is independent of mean expression level. PseudoGA shows consistent behavior across all three scenarios.

We present boxplots of the two criteria for all methods under consideration. Results of our simulation study indicate that our PseudoGA shows superior performance compared to other methods for the first two scenarios while for the third its accuracy is at least as good as other methods (Figure 9, Figure 10). Thus PseudoGA looks promising in identifying pseudotime trajectory in a variety of situations for single cell data.

Our simulation method is very general and has very little (or no) bearing with PseudoGA. However, to see its performance in wider scenarios, we also simulate expression data using Bioconductor package ‘splatter’ under three different methods: PROSSTT (56), Splat (57) and PhenoPath (43). In each dataset generated by Splat, the expression values of the gene with the highest variance were permuted randomly among the corresponding cells. This helps in assessing the performances of different methods on datasets with selected genes containing few misspecified genes or few genes that behave like outliers from the rest. Since PhenoPath simulates log-normalized expressions, anti-log transformation was applied on the data before benchmarking with different methods. Lognormal assumption generates genes with very high variance in expression values. Simulations with Splat and PROSSTT were performed with 300 cells and 100 genes.

To verify whether our subsampling based approach performs comparably with other methods, we simulate 3000 cells and100 genes using PhenoPath under the third scenario. Details of the simulation procedure is described in Supplementary Section 2.

**Figure 11.**
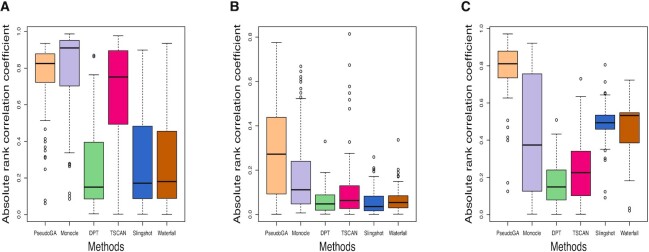
Absolute correlation coefficient with the actual pseudotime in simulations with (**A**) PROSSTT, (**B**) Splat and (**C**) PhenoPath. PseudoGA performs best in (B) and (C).

**Figure 12.**
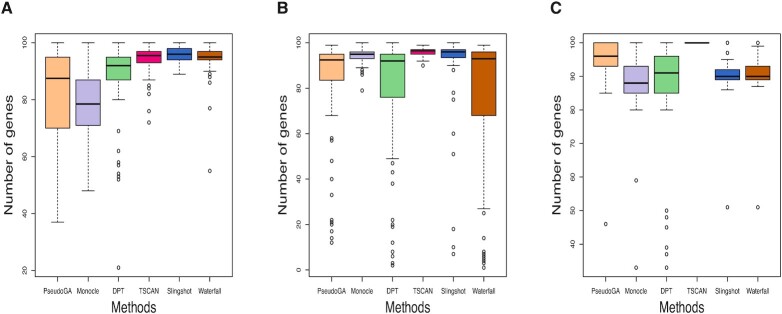
Number of genes having functional relationship with pseudotime estimated in simulations with (**A**) PROSSTT, (**B**) Splat and (**C**) PhenoPath. PseudoGA performance is consistent across all three simulations.

Accuracies of different methods were compared using the same two criteria as before. Absolute rank correlation coefficient is a measure of concordance between estimated pseudotime and the actual pseudotime, whereas number of functionally related genes is an evidence based measure of concordance. The performance of PseudoGA is consistent across all three situations (Figure 11, Figure 12). Monocle marginally outperformed PseudoGA in PROSSTT simulation. In Splat simulation, even with only one perturbed gene, accuracy of all methods except PseudoGA is downgraded due to the presence of uncorrelated genes with high variance. In PhenoPath simulation, where the distribution of gene expression differs from usual negative binomial assumption, PseudoGA turns out to be more robust than other methods. Thus, performance of PseudoGA under PhenoPath and Splat simulation indicates that it maintains its accuracy and robustness in presence of outliers and highly variable genes.

We also check the performance of PseudoGA in case of a large dataset and effectiveness of the subsampling based approach. We simulate a trajectory with 10 000 cells with expression values following Gamma-Poisson distribution and construct trajectory using PseudoGA with only $1\%$ of the data. The remaining cells are added afterwards using nearest neighbour approach as proposed in Material and Methods Section. Based on 30 replications, the median absolute correlation between the actual pseudotime and the pseudotime generated by 100 cells was found to be 0.85 whereas the median absolute correlation with all 10 000 cells was found to be 0.98. The comparison between the actual pseudotime and the estimated pseudotime based on one subsample is shown in Supplementary Figure S24.

### Scalability

Runtime of any genetic algorithm depends on population size in each generation and the number of generations. In general, increasing the values of these parameters will improve the accuracy of an algorithm and at the same time will increase the runtime. In this article, the cost function has been evaluated on 400 permutations in each generation and a minimum of 30 generations have been considered in all simulation and real data analyses. The value of ε was taken to be a pre-assigned small positive number. Different algorithms scale differently with number of cells and number of features (58). PseudoGA approximately scales the same linearly. Using subsampling based approach, we have proposed a method, to tackle the increasing volume of single cell data with large number of cells.

**Figure 13.**
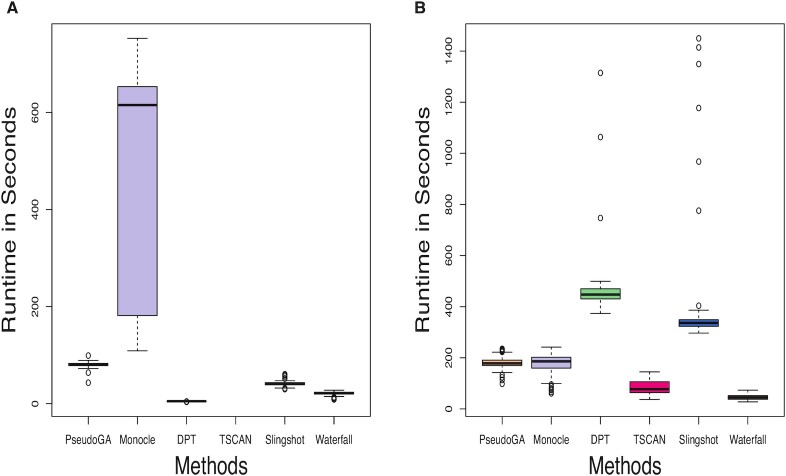
Runtime for different algorithms with (**A**) 300 cells and 10 000 features (**B**) 3000 cells and 1000 features. PseudoGA runtime is comparable with other methods. Because of subsampling approach PseudoGA gains in runtime efficiency in (B).

To assess scalability of PseudoGA, we benchmark PseudoGA runtime against runtime of other methods. We consider two types of count data generated by Splatter: one with 300 cells and 10 000 features and the other with 3000 cells and 1000 features. In the second scenario, we run PsedoGA with three subsamples each of size 100 coupled with nearest neighbor matching and principal curve fitting. The boxplots of the runtimes based on 100 replicates are shown in Figure 13. For large number of cells, PseudoGA gains time efficiency by using subsampling approach (Figure 13B). Since pseudotime estimation on subsamples can be performed independently, parallelization with respect to different subsamples leads to further time efficiency of this approach. Figure 13 indicates that PseudoGA is time efficient both with respect to a large number of genes as well as a large number of cells.

## DISCUSSION

Several algorithms have been designed to order cells along pseudotime trajectory based on single cell RNA-seq data. These are mainly based on the philosophy of constructing trajectory on reduced dimensional data. Some genes might show distinct types of functional pattern of expression levels along different transcriptomic stages. Hence, it may be possible that information on gene expression level might be lost to some extent, sometimes substantially, during the dimensionality reduction step. This loss of information may lead to erroneous outcome at the next step while inferring the ordering of cells. Entire pseudotime construction depends on the amount of information captured in reduced dimensionality of data. Moreover, few genes may remain approximately constant over the entire time trajectory, whereas few may be outliers. Presence of such genes might influence the pseudotime construction. We devise an algorithm ‘PseudoGA’ that searches for the best possible ordering of cells in the set of all permutations and infers the ordering based on actual gene expression levels.

Our proposed method PseudoGA assumes that the dependency structure of gene expression on pseudotime is based on ranks of its values. This allows our method to encompass a large class of functions that gene expression values can assume along a trajectory. To tackle with zeros, we accommodate average ranking to all cells with zero expressions for a given gene. This nonparametric assumption makes our method robust in different types of single cell expression datasets.

We first cluster the cells in homogeneous subgroups and apply genetic algorithm on each of these homogeneous groups to increase the efficiency, followed by a novel method to concatenate paths from different clusters. This will help us in identifying any lineage or branching structure that may exist in the data with respect to pseudotime. Otherwise, we can always apply our algorithm directly to the entire dataset.

Compared to other existing methods, PseudoGA seems more robust when applied on various real datasets as well as on simulated datasets. PseudoGA has been shown to be consistent in various simulation schemes. In presence of outliers or highly variable genes, methods based on dimensionality reduction could fail but PseudoGA maintains its accuracy and robustness.

Our proposed method can be applied to a variety of datasets with even large number of cells. Our study reveals that even in such a situation, the performance of PseudoGA is extremely well both in terms of accuracy and time. We speculate that further improvements on scalability of the method are possible by implementing a more efficient genetic algorithm and parallelization of the code in case of large datasets. One can use more operators in addition to the three operators used in PseudoGA and apply them in different manners. Improvement in the genetic algorithm would certainly improve the efficiency of PseudoGA. To the best of our knowledge, this is probably the first application of genetic algorithm in pseudotime estimation with some novel ideas and methods inbuilt in the main algorithm. PseudoGA is a freely available software implemented in R and can conveniently be applied on any single cell expression data.

## Supplementary Material

gkab457_Supplemental_File
