# Supplementary material for: PseudoGA: cell pseudotime reconstruction based on genetic algorithm
Source: Nucleic Acids Res. 2021 Jul 9;49(14):7909–24. doi: 10.1093/nar/gkab457 (PMC8661435; doi:10.1093/nar/gkab457)
Supplement: gkab457_Supplemental_File [file gkab457_supplemental_file.pdf]

PseudoGA: Cell pseudotime reconstruction based on genetic algorithm

Pronoy Kanti Mondal , Udit Surya Saha and Indranil Mukhopadhyay*

Human Genetics Unit, Indian Statistical Institute,
203 B. T. Road, Kolkata 700108, West Bengal, India
**Corresponding author*

1 Our algorithm for data simulation

We assume that read counts follow Gamma-Poisson distribution and we allow the parameters of the Gamma distribution to change with pseudotime. Let us assume that there are n cells with ordering $i_1, i_2, ..., i_n$ where $(i_1, i_2, ..., i_n)$ is a permutation of $(1, 2, ..., n)$. The following data generation model has been used for simulating read counts from a set of genes:

Step 1:

We construct $\lambda_{kj} = \alpha_j + \beta_j k$ which is the shape parameter of the Gamma distribution in the k-th cell for j-th gene, α_j and β_j are gene specific parameters. For any given gene with index j, λ_{kj} changes with the position of cells on the pseudotime trajectory and exhibits linear trend along pseudotime ordering. Next, we simulate X_{kj} where $X_{kj} \sim Gamma(\lambda_{kj}, r_j)$, and λ_{kj} is the shape parameter and r_j is a gene specific scale parameter. Then, we generate Z_{kj} that indicates whether j-th gene is expressed in k-th cell or not. We assume $Z_{kj} \sim Bernoulli(\Phi(c_j + \alpha X_{kj}))$, c_j is a gene specific constant, $\alpha \geq 0$. Note that, in scenario I, the probability that a gene is expressed is an increasing function of the Gamma variable X_{kj} ($\alpha > 0$) whereas in scenarios II and III, they are independent ($\alpha = 0$). Finally, we generate $(Y_{kj}|Z_{kj} = 0) = 0$ and

$(Y_{kj}|Z_{kj} = 1) \sim Poisson(X_{kj})$ where Y_{kj} denotes the read count of j-th gene in k-th cell. Let $Y_{.j} = (Y_{1j}, \ldots, Y_{nj})$.

Step 2:

After Step 1, the set of expression values for any particular gene has increasing linear trend along pseduotime trajectory if β_j is taken positive. If we want j-th gene to have decreasing mean expression level with pseudotime, we modify the set of expression values for j-th gene as $Y'_{.j} = \text{reverse}(Y_{.j})$ where $\text{reverse}(x_1, x_2, ..., x_n) = (x_n, x_{(n-1)}, ..., x_1)$. To include genes with both increasing and decreasing trend, we apply $Y'_{.j} = \text{reverse}(Y_{.j})$ for some genes and keep $Y'_{.j} = Y_{.j}$ for the rest.

Step 3:

To construct a gene with approximate quadratic trend with respect to pseudotime, we modify $Y'_{.j}$ from Step 2 in the following manner:

$$Y''_{.j} = Y'_{.j}[1, 3, 5, ..., (2n-1), (2n), (2n-2), ..., 2]$$

To construct a gene with approximate cubic trend or sinusoidal trend with respect to pseudotime, we modify $Y'_{.j}$ in the following manner:

$$u = (1, 3, 5, ..., (2n-1))$$
$$v = (2n, (2n-2), (2n-4), ..., 2)$$
$$Y''_{.j} = Y'_{.j}[u[(\tfrac{n}{2}+1) : n], v, u[1 : (\tfrac{n}{2})]]$$

To generate genes that are not associated with pseudotime, we take $Y''_{.j}$ as a random permutation of $Y'_{.j}$.

To include variety of trends in our final gene expression matrix, we consider four types of genes: genes with quadratic trend, genes with cubic trend, genes with linear trend from Step 2 that we keep unchanged in Step 3 and genes with no association with pseudotime. In scenarios I and II, we assume relatively high values of $c_j (c_j \sim N(1, \frac{1}{4}))$ implying lower occurrence of zeros. In scenario III, relatively lower values of $c_j (c_j \sim N(0, \frac{1}{4}))$ are considered to incorporate higher incidence of dropouts.

The expression values for the j-th gene corresponding to the ordering $(i_1, i_2, ..., i_n)$, $s_{.j}$ is obtained by equating $(s_{i_1 j}, s_{i_2 j}, ..., s_{i_n j}) = Y''_{.j}$

2 Simulation with Splatter

Using the Bioconductor package Splatter (1), single cell RNA-seq data were generated using three different methods: PROSSTT (2), Splat (1) and PhenoPath

(3). Under PROSSTT simulation method, the datasets of the specified size were generated with 100 steps keeping all other parameters as default.

Similarly, datasets with the specified number of features and cells were generated by Splat simulation method with 100 steps keeping all other parameters as default. Let i-th gene has the highest variance of expression values. The expression values of the i-th gene X_i is changed into $\sigma(X_i)$ where $\sigma(.)$ represents a random permutation of $(1, 2, \ldots, n)$, n being the number of cells.

Similarly, simulations using PhenoPath with the specified size were performed with all default parameters. PhenoPath generates expression values comparable to log normalized expressions. To convert them into actual expression values, the following transformation is applied on expression vectors X_i for the i-th gene: $Y_i = 10^{X_i - min(X_i) - 3}$. Y_i values for all 100 genes are used for estimation by different methods. The distribution of Y_i can be thought of as left truncated unimodal log-normal, which is known to be a good approximation of normalized single cell expression data (4).

3 Operators in genetic algorithm

We explain different operators like mutation, recombination and selection that are used in our algorithm. We explain these using two chromosomal representations of eight objects as given in Figure S1.

**Chromosome A**	7 6 5 1 2 3 4 8
**Chromosome B**	6 1 4 2 5 7 3 8

Figure S1: Two different chromosomal representations of permutations.

Mutation operator, when applied on one permutation, arranges the objects in the segment between two positions in reverse order (Figure S2). We can also think of similar kinds of other mutation operators.

We apply recombination operator on two parental chromosomal configurations. This would produce two new offspring chromosomes that are different from parental chromosomes (Figure S3).

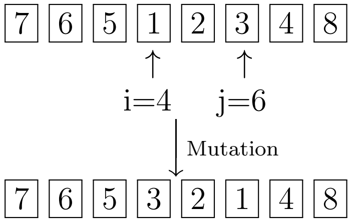

Figure S2: Mutation with the segment between position 4 to 6 being reversed.

Parent 1 | | Parent 2

7 6 5 1 | 2 3 4 8 + 6 1 4 2 | 5 7 3 8

Crossover point | | Crossover point

↓ Recombination

Offspring 1 | | Offspring 2

7 6 5 1 U U U U + U U U U 5 7 3 8

Offspring 1

Missing entries in string 1 | | Missing entries from string 1 in string 2

O O O O 2 3 4 8 + X X 4 2 X X 3 8

O: Occupied positions
U: Unoccupied positions
X: Ignored positions

Position of missing entries in string 1 | | Position of missing entries from string 1 in string 2

O O O O U U U U + X X □ □ X X □ □

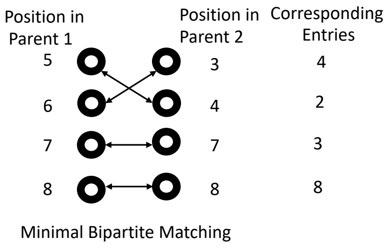

↓ Offspring 1

7 6 5 1 2 4 3 8

Offspring 2

Missing entries in string 2

6 1 4 2 O O O O

Missing entries from string 2 in string 1

\+ X 6 X 1 2 X 4 X

Position of missing entries in string 2

U U U U O O O O

Position of missing entries from string 2 in string 1

\+ X _ X _ _ X _ X

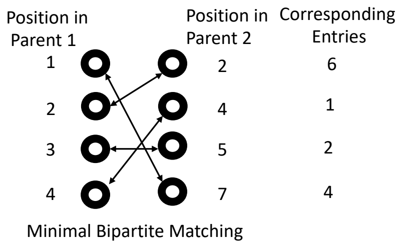

↓ Offspring 2

4 6 2 1 5 7 3 8

Result of recombination

↓

Offspring 1 | Offspring 2

7 6 5 1 2 4 3 8 + 4 6 2 1 5 7 3 8

Figure S3: Recombination with breakpoint between position 4 and 5

If there are N chromosomal configurations, recombination produces another N new offspring configurations making a total of $2N$ configurations. Again applying mutation operator we get $4N$ configurations (permutations). Now we apply selection operator (Figure S4) to select top N permutations based on the chosen cost function.

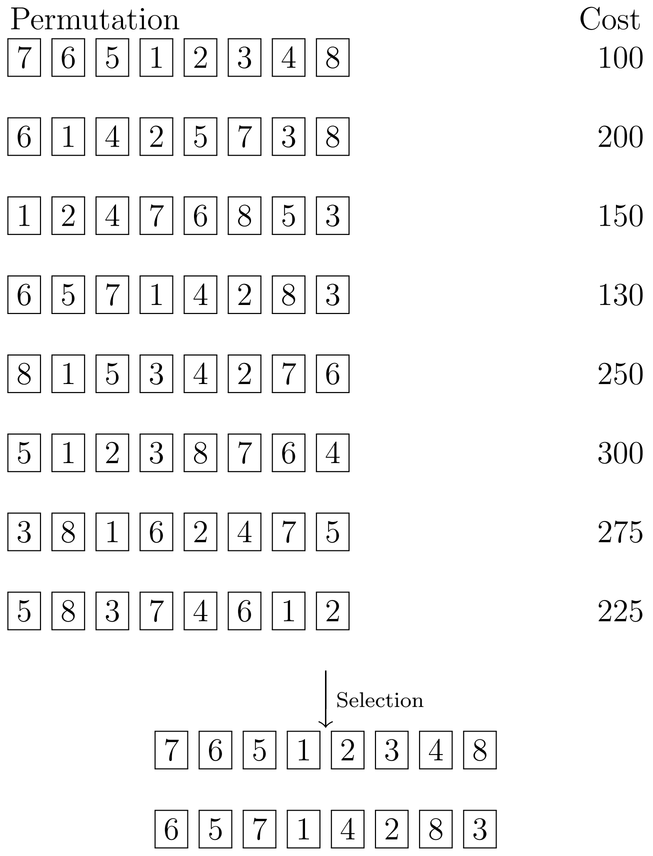

Figure S4: Selection with 8 permutations

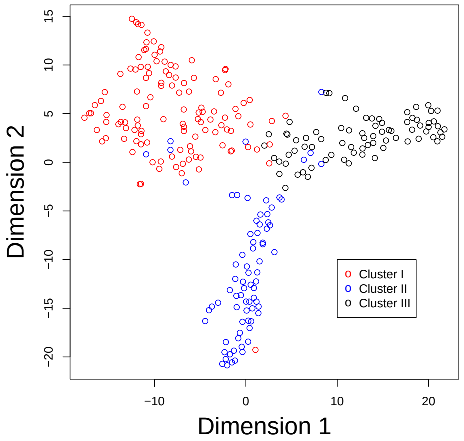

Figure S5: t-SNE plot of the HSMM data with memberships obtained from k-means clustering.

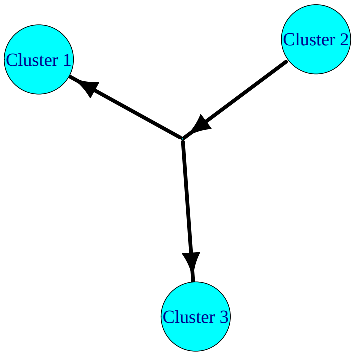

Figure S6: Network between clusters in HSMM data estimated by PseudoGA.

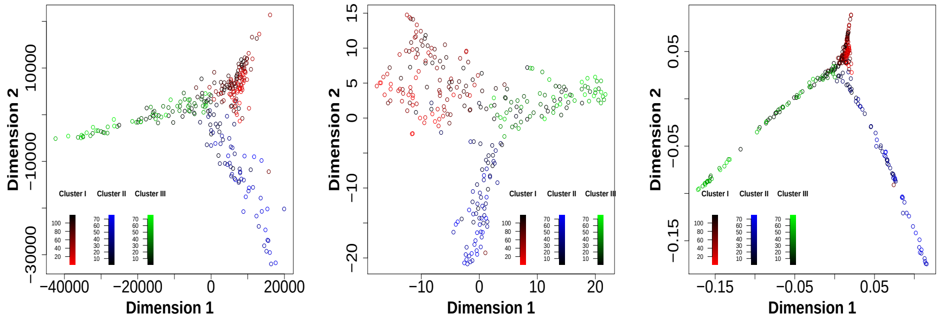

(A) **(B)** **(C)**

Figure S7: Visualization of PseudoGA ordering with **(A)** PCA, **(B)** t-SNE, and **(C)** Diffusion Map, using HSMM dataset. Clustering was performed using k-means clustering on reduced dimensional data obtained using t-SNE. We calculate 23 different indices and choose the optimum number of clusters by majority voting. This can be done using R package "NbClust"(5).

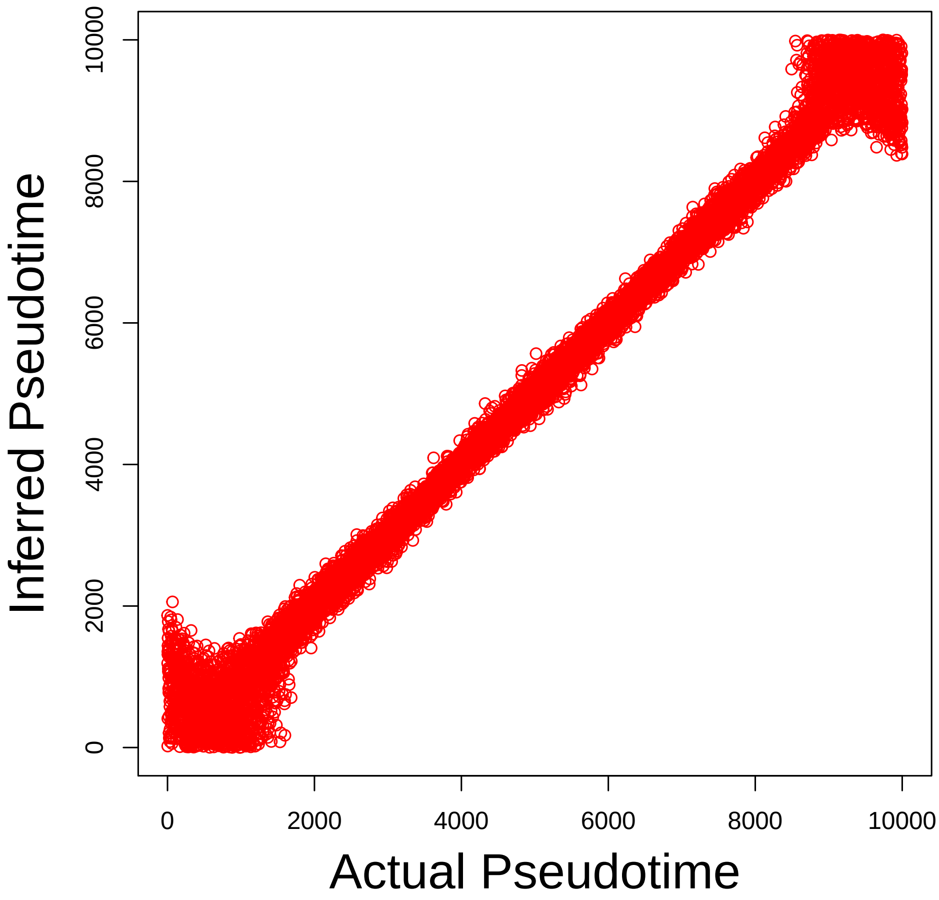

Figure S8: Pseudotime ordering by PseudoGA with multiple subsamples based on large data.

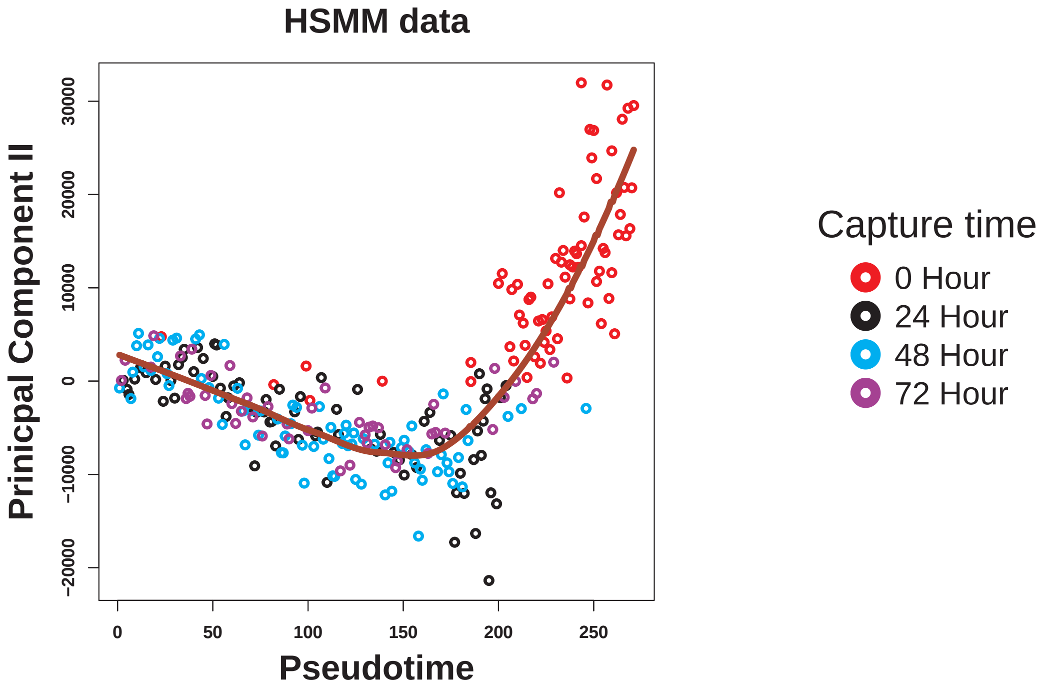

Figure S9: Plot of second principal component on the whole data with pseudotime estimated by PseudoGA. PC II shows quadratic pattern with pseudotime estimated by PseudoGA.

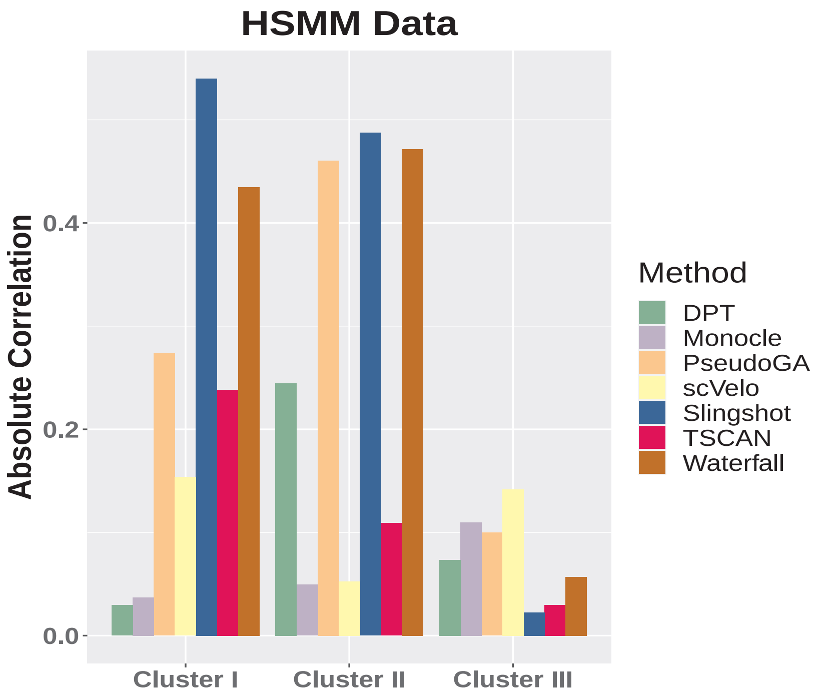

Figure S10: Absolute Spearman's rank correlation between HSMM data capture time and pseudotime produced by different methods. PseudoGA remains among top three methods across all three clusters.

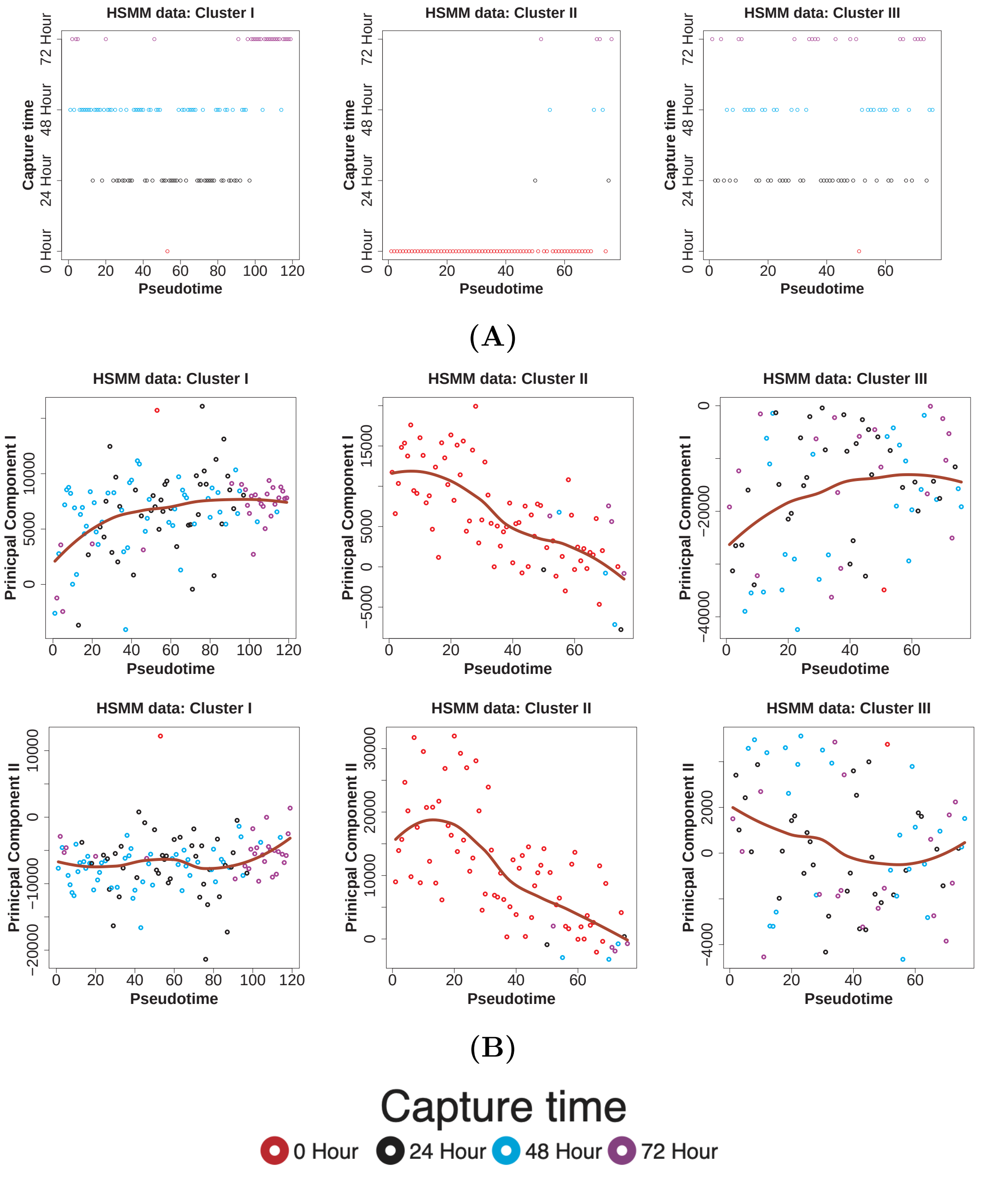

Figure S11: (A) Pseudotime ordering by PseudoGA on HSMM dataset on three different clusters (B) Functional relationship between first two principal components and pseudotime estimated by PseudoGA on three different clusters. In all cases, the relationship is either linear or quadratic.

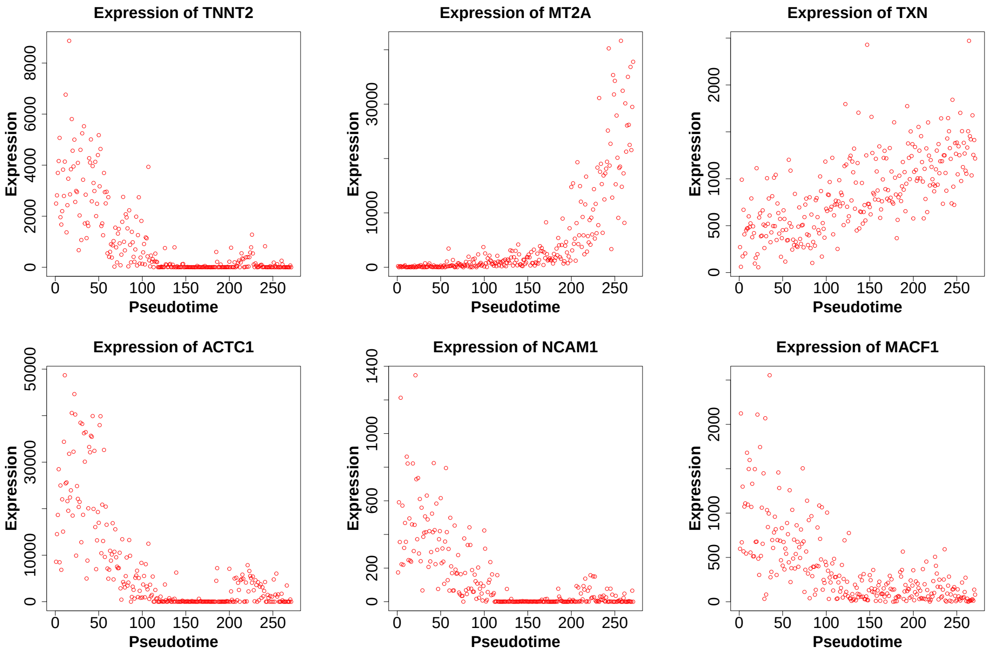

Figure S12: Change of expression pattern with pseudotime in HSMM dataset showing top 6 genes with highest correlation on the entire dataset.

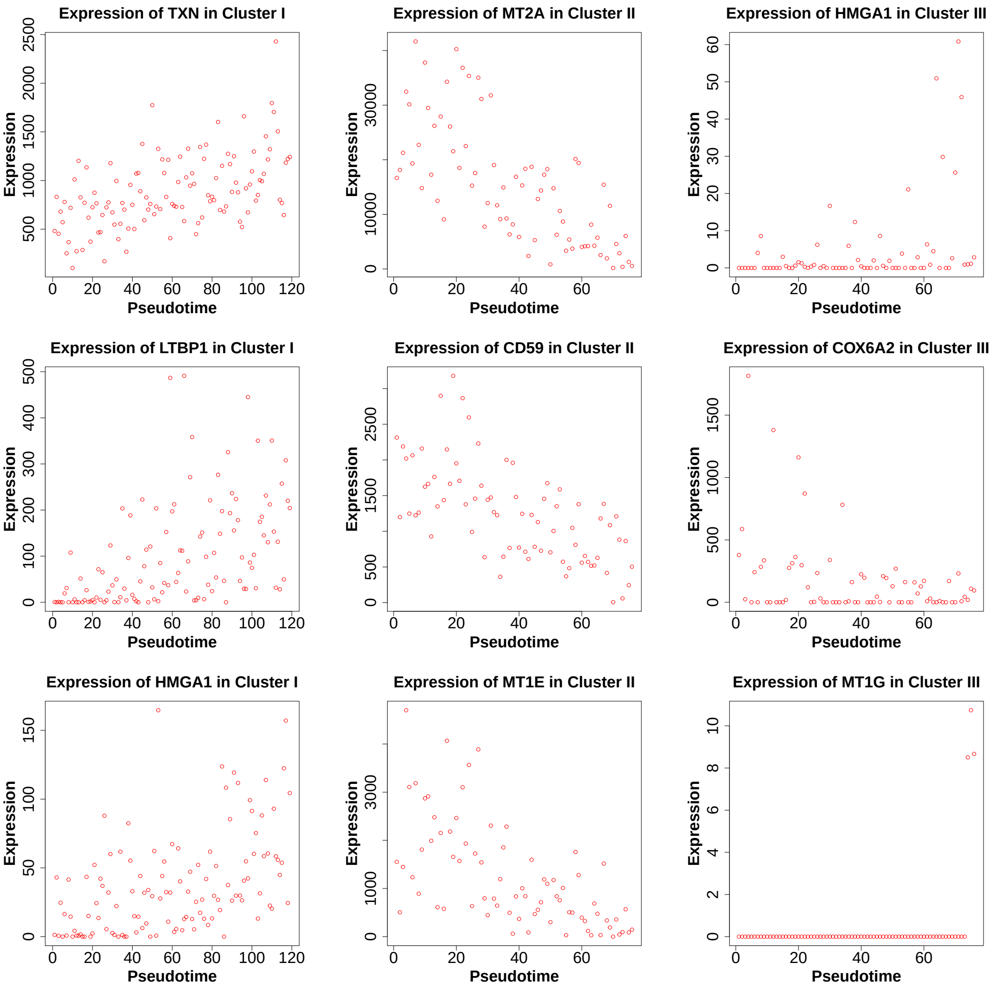

Expression of TXN in Cluster I
Expression of MT2A in Cluster II
Expression of HMGA1 in Cluster III
Expression of LTBP1 in Cluster I
Expression of CD59 in Cluster II
Expression of COX6A2 in Cluster III
Expression of HMGA1 in Cluster I
Expression of MT1E in Cluster II
Expression of MT1G in Cluster III
Expression
Pseudotime

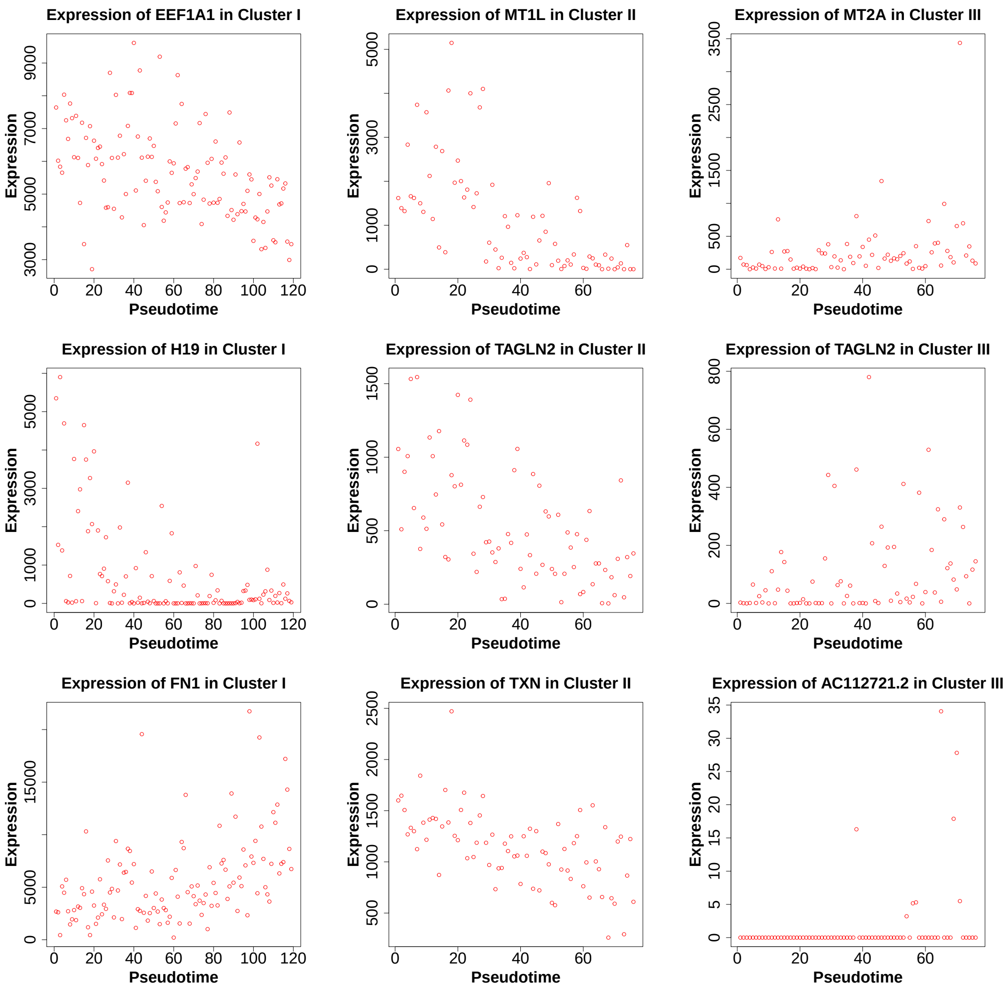

Figure S13: Change of expression pattern with pseudotime in HSMM dataset showing top 6 genes with highest correlation in each of the three clusters.

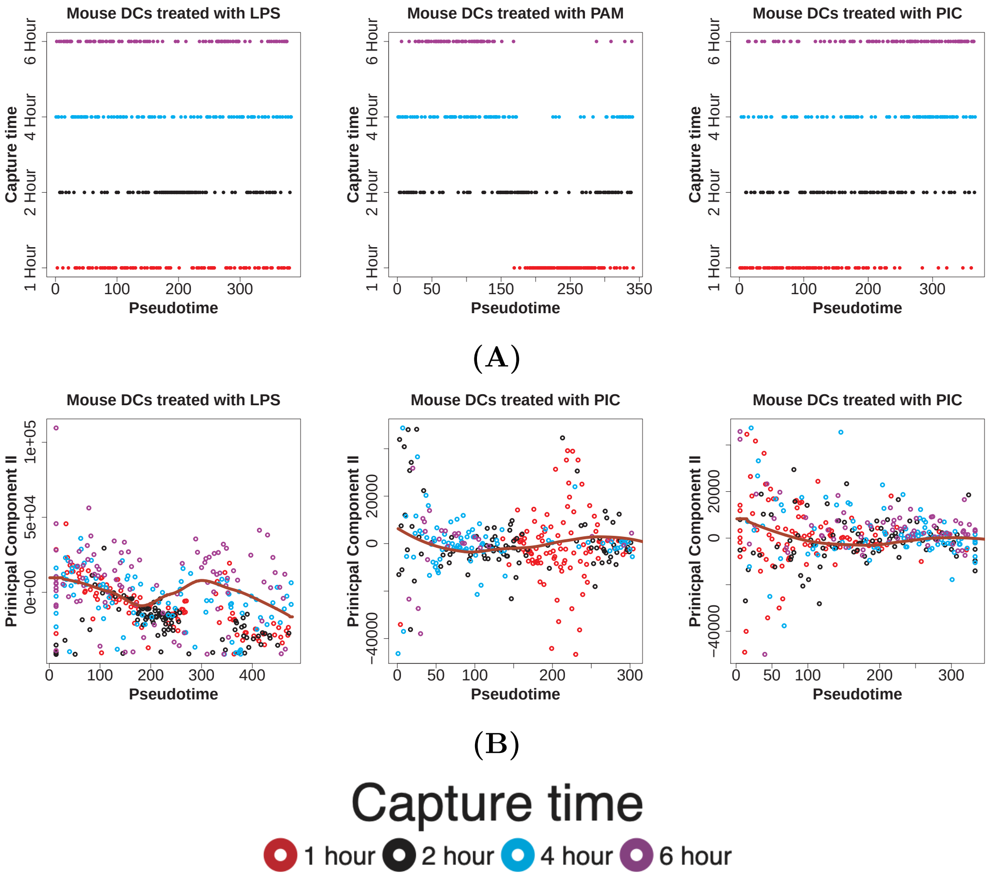

Figure S14: (A) Pseudotime estimated by PseudoGA on the dataset where mouse dendritic cells are treated with LPS, PAM and PIC respectively. (B) Functional relationship between PC II and the pseudotime estimated by PseudoGA on the same datasets.

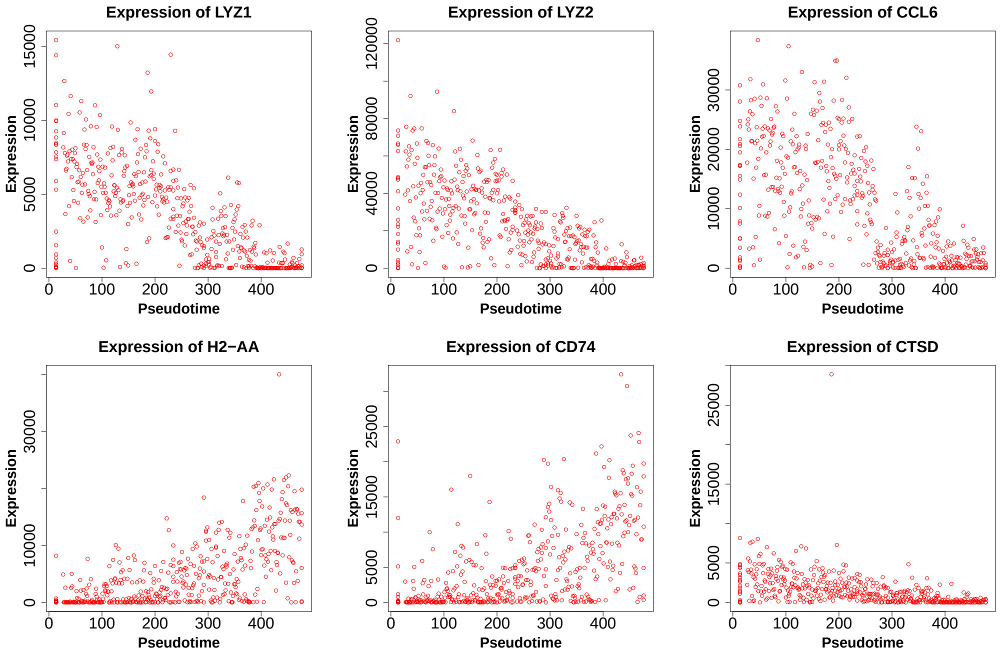

Figure S15: Change of expression pattern with pseudotime when dendritic cells are treated with LPS stimulus showing top 6 genes with highest correlation.

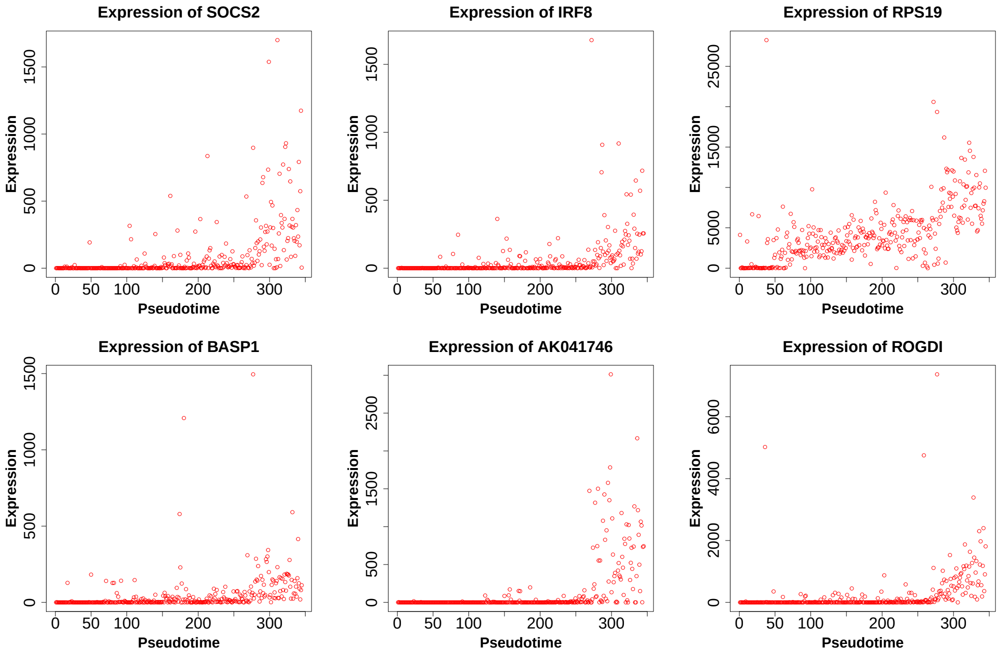

Figure S16: Change of expression pattern with pseudotime when dendritic cells are treated with PAM stimulus showing top 6 genes with highest correlation.

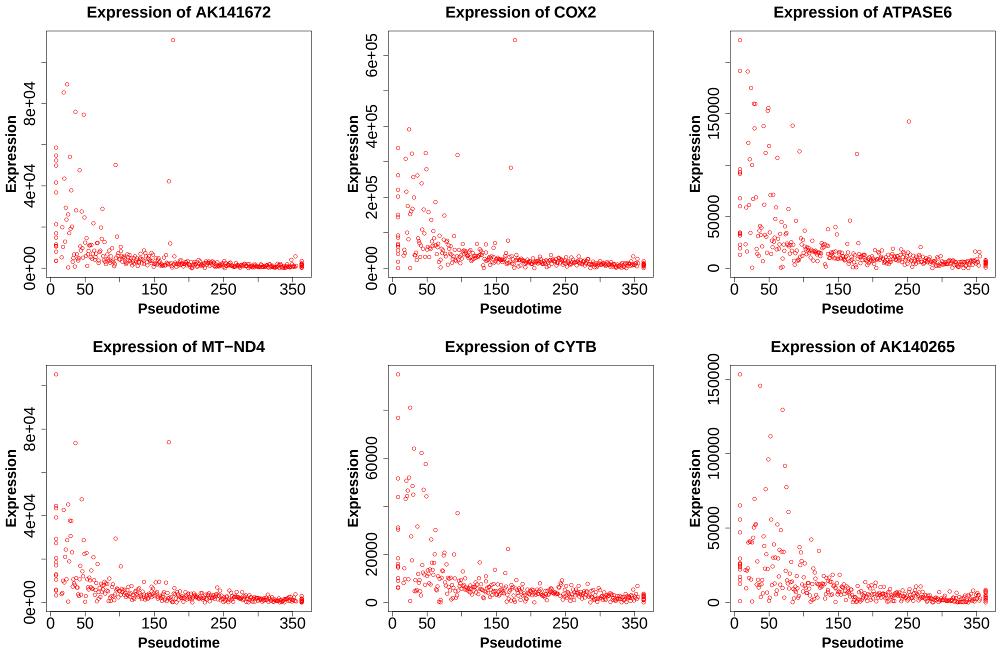

Figure S17: Change of expression pattern with pseudotime when dendritic cells are treated with PIC stimulus showing top 6 genes with highest correlation.

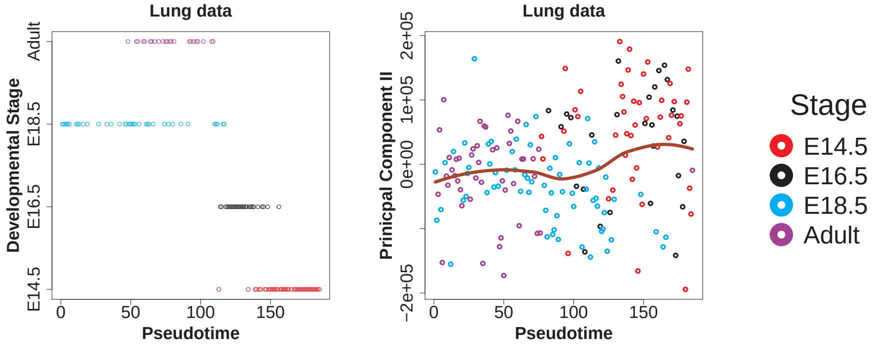

Figure S18: Pseudotime ordering by PseudoGA on mouse lung dataset and the functional relationship between PC II and the pseudotime estimated. The plot shows monotonic pattern of PC II with pseudotime.

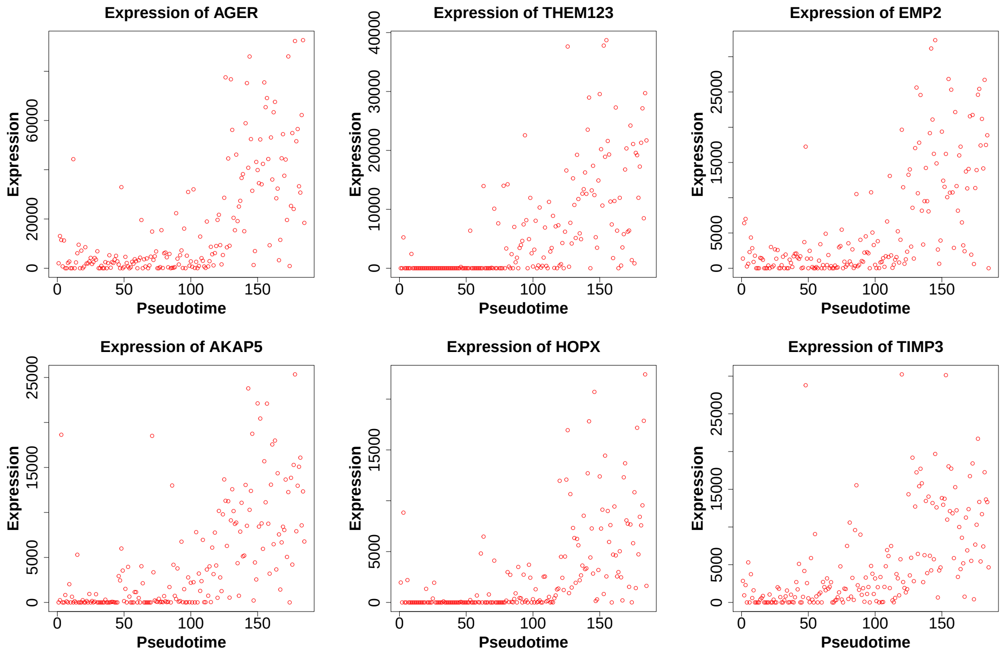

Figure S19: Change of expression pattern with pseudotime in mouse lung dataset showing top 6 genes with highest correlation.

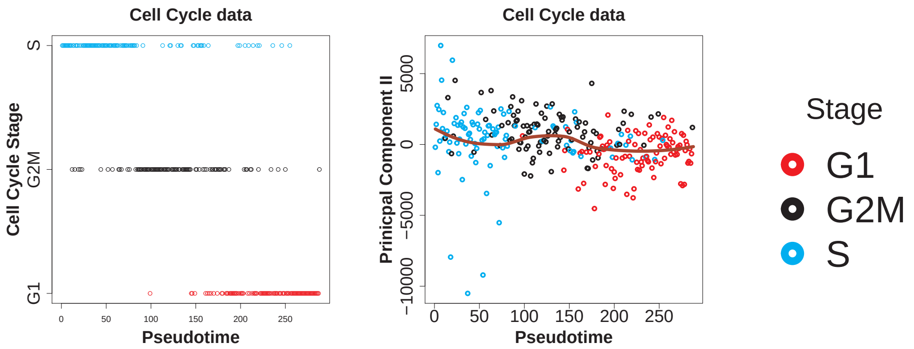

Figure S20: Pseudotime ordering by PseudoGA on mouse cell cycle dataset and the functional relationship between PC II and the pseudotime estimated. PC II shows linear pattern with the pseudotime estimated.

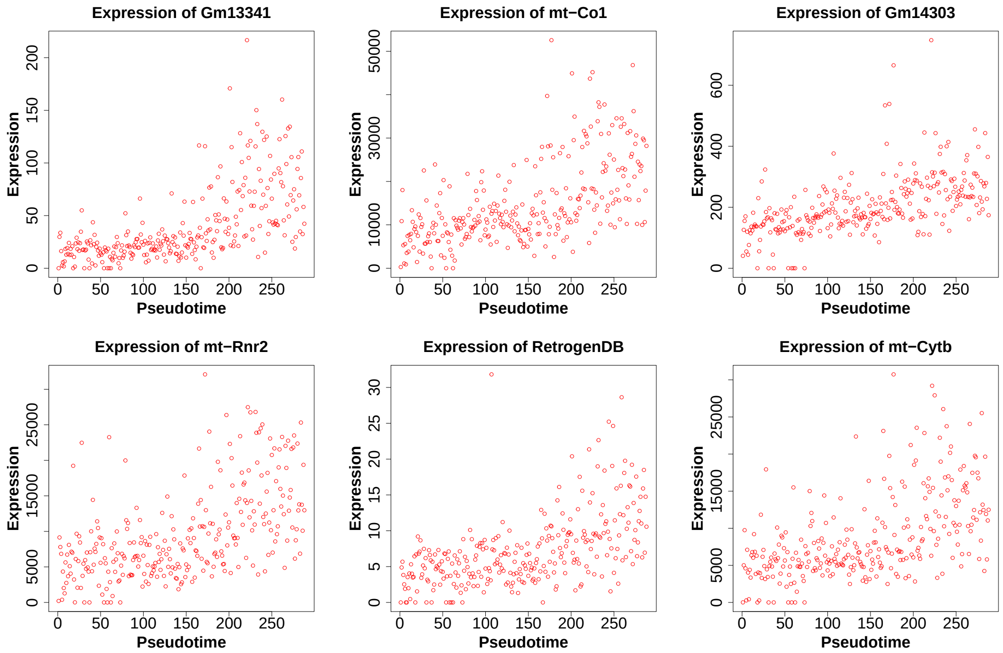

Figure S21: Change of expression pattern with pseudotime in mouse cell cycle dataset showing top 6 genes with highest correlation.

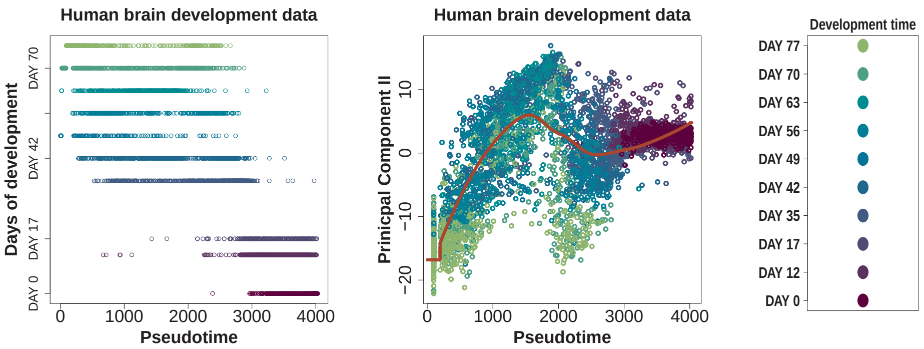

Figure S22: Pseudotime ordering by PseudoGA on human brain development dataset and the functional relationship between PC II and the pseudotime estimated. PC II shows cubic relationship with the estimated pseudotime.

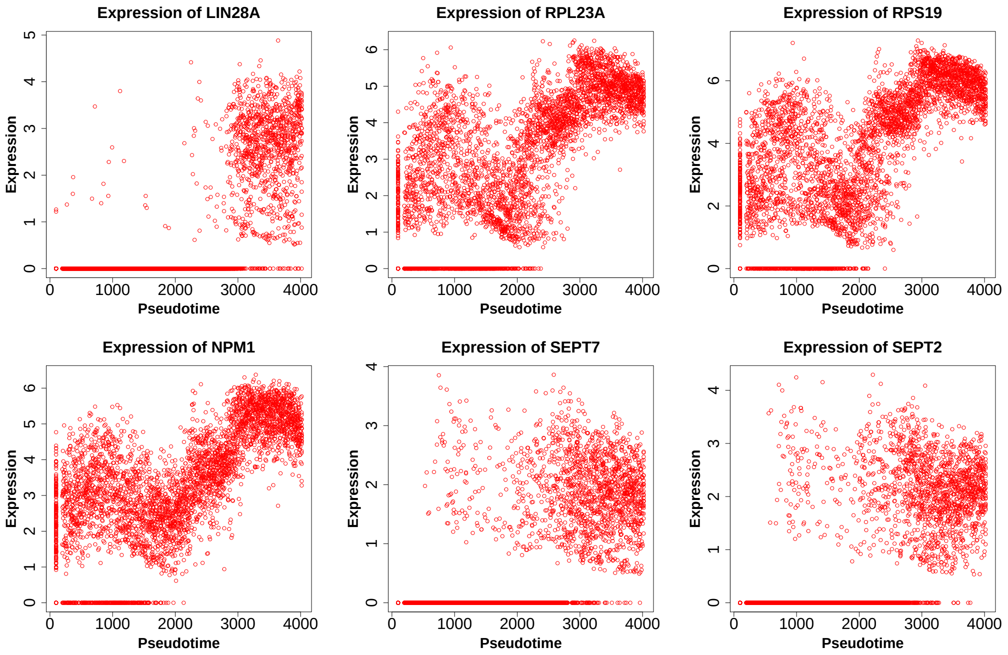

Figure S23: Change of expression pattern with pseudotime in human brain development dataset showing top 6 genes with highest correlation. These genes show either cubic or bursting type patterns with the estimated pseudotime.

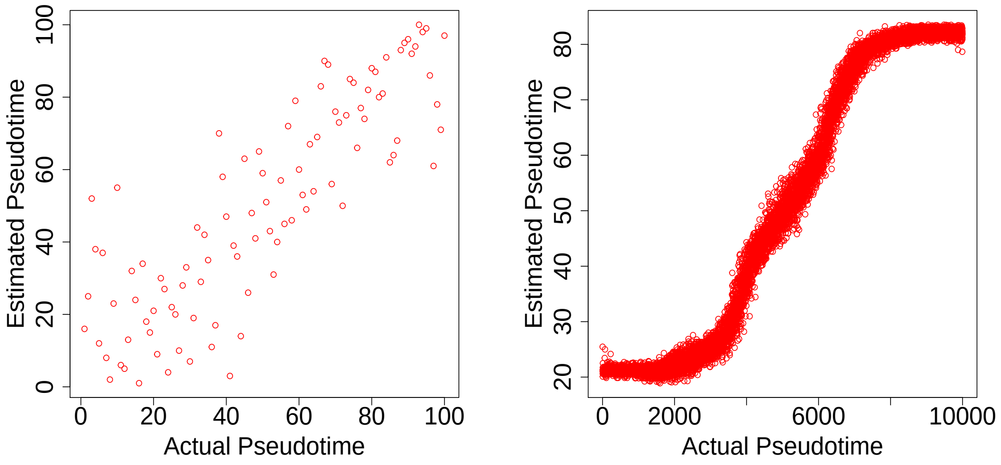

(A) **(B)**

Figure S24: **(A)** An instance of pseudotime ordering by PseudoGA with a subset of cells and **(B)** Pseudotime ordering of all cells with our suggested algorithm for large data based on the same subset.

Cluster id	Genes with highest correlation	Common function
Entire dataset	TNNT2, MT2A, TXN, ACTC1, NCAM1, MACF1	Muscle functioning

Table S1: Top 6 genes having highest correlation with pseudotime estimated by PseudoGA ignoring clusters and their common function in the HSMM dataset.

Cluster id	Genes with highest correlation	Common function
Cluster I	TXN, LTBP1, HMGA1, EEF1A1, H19, FN1	Oxidative stress
Cluster II	MT2A, CD59, MT1E, MT1L, TAGLN2, TXN	Oxidative stress
Cluster III	HMGA1, COX6A2, MT1G, MT2A, TAGLN2, AC112721.2	Heme binding

Table S2: Top 6 genes having highest correlation with pseudotime estimated by PseudoGA in each cluster and their common functions in HSMM dataset.

Cells treated with LPS		
Cluster id	Genes with highest correlation	Common function
Entire dataset	LYZ1, LYZ2, CCL6, H2-AA, CD74, CTSD	Immune response

Table S3: Top 6 genes having highest correlation with pseudotime estimated by PseudoGA and their common function in dendritic cells dataset with LPS stimulus.

Cells treated with PAM		
Cluster id	Genes with highest correlation	Common function
Entire dataset	SOCS2, IRF8, RPS19, BASP1, AK041746, ROGDI	Apoptosis(except AK041746)

Table S4: Top 6 genes having highest correlation with pseudotime estimated by PseudoGA and their common function in dendritic cells dataset with PAM stimulus.

Cells treated with PIC		
Cluster id	Genes with highest correlation	Common function
Entire dataset	AK141672, COX2, ATPASE6, MT-ND4, CYTB, AK140265	Mitochondrial functions

Table S5: Top 6 genes having highest correlation with pseudotime estimated by PseudoGA and their common function in dendritic cells dataset with PIC stimulus.

Cluster id	Genes with highest correlation	Common function
Entire dataset	AGER, THEM123, EMP2, AKAP5, HOPX, TIMP3	Lung functioning

Table S6: Top 6 genes having highest correlation with pseudotime estimated by PseudoGA and their common function in mouse lung dataset.

Cluster id	Genes with highest correlation	Common function
Entire dataset	Gm13341(Pseudogene), mt-Co1(mtRNA), Gm14303(Pseudogene), mt-Rnr2(mtRNA), RetrogenDB(Pseudogene), mt-Cytb (mtRNA)	Unknown

Table S7: Top 6 genes having highest correlation with pseudotime estimated by PseudoGA and their common function in mouse cell cycle dataset.

Cluster id	Genes with highest correlation	Common function
Enntire dataset	LIN28A, RPL23A, RPS19, NPM1, SEPT7, SEPT2	Development

Table S8: Top 6 genes having highest correlation with pseudotime estimated by PseudoGA and their common function in human brain development dataset.

References

[1] Zappia,L., Phipson,B. and Oshlack,A. (2017). Splatter: simulation of single-cell RNA sequencing data. *Genome Biology*, **18:174**.

[2] Papadopoulos,N., Gonzalo,P.R. and Soding,J. (2019). PROSSTT: probabilistic simulation of single-cell RNA-seq data for complex differentiation processes. *Bioinformatics*, **35(18)**, 3517–3519.

[3] Campbell,K.R. and Yau,C. (2018) Uncovering pseudotemporal trajectories with covariates from single cell and bulk expression data. *Nature Communications*, **9:2442**.

[4] Wan,C., Chang,W., Zhang,Y., Shah,F., Lu,X., Zang,Y., Zhang,A., Cao,S., Fishel,M.L. and Ma,Q. et al. (2019) LTMG: a novel statistical modeling of transcriptional expression states ins single-cell RNA-seq data. *Nucleic Acids Research*, **47(18):e111**.

[5] Charrad,M., Ghazzali,N., Boiteau,V. and Niknafs,A. (2014). NbClust: An R Package for Determining the Relevant Number of Clusters in a Data Set. *Journal of Statistical Software*, **61(6)**.
